# Lake sediment heatwaves under global warming

**DOI:** 10.1038/s41561-026-01986-3

**Published:** 2026-06-01

**Authors:** R. Iestyn Woolway, Haoran Shi, Zeli Tan, Joachim Jansen

**Affiliations:** 1 School of Ocean Sciences, Bangor University, Menai Bridge, Wales; 2 Atmospheric, Climate and Earth Sciences Division, Pacific Northwest National Laboratory, Richland, WA USA; 3 Institute for Atmospheric and Earth System Research, University of Helsinki, Helsinki, Finland

**Keywords:** Limnology, Biogeochemistry, Climate change

## Abstract

Lake sediment heatwaves, driven by rising global temperatures, pose emerging threats to freshwater ecosystems by altering sediment thermal regimes and intensifying sediment biogeochemical processes. Here we present a global-scale assessment of lake sediment heatwaves, examining their historical patterns and projecting future trends under various climate scenarios in 41,499 representative lakes worldwide. Using daily simulated lake sediment temperatures from 1981 to 2010 and future (2071–2100) projections under three Shared Socioeconomic Pathways (SSP 1–2.6, SSP 3–7.0, SSP 5–8.5), we investigate lake sediment heatwave characteristics, including their duration, intensity, frequency and seasonal timing worldwide. Our results show that lake sediment heatwaves are generally more persistent and frequent than lake surface heatwaves, with sediment heatwaves in pelagic regions experiencing a notable lag relative to surface conditions. Under future climate scenarios, sediment heatwaves are projected to intensify, with their duration and frequency increasing substantially, particularly under SSP 5–8.5. These shifts could exacerbate the production of greenhouse gases such as methane and increase sediment respiration rates in lakes. This study highlights the need to account for sediment heatwaves in freshwater ecosystem management and climate adaptation strategies to mitigate future impacts.

## Abstract (web summary)

Model analyses and projections of sediment heatwave duration, intensity and frequency across 41,499 representative lakes suggest that lake sediment heatwaves are more persistent and frequent than lake surface heatwaves and will intensify under global warming.

## Main

Heatwaves, defined as prolonged periods of anomalously high temperatures, are among the most impactful consequences of global warming, with far-reaching ecological, societal and economic implications^1–3^. Whereas atmospheric heatwaves have been extensively studied^4–6^, recent research has increasingly highlighted the occurrence and impacts of heatwaves in aquatic environments, particularly under global warming^7–9^. Marine heatwaves, which are defined as prolonged periods of anomalously warm sea surface temperatures^7,10,11^, have been extensively studied due to their wide-ranging ecological impacts on marine ecosystems^12,13^. In contrast, heatwaves in freshwater systems, particularly lakes, have only recently gained attention^8,14–16^ but have been shown to considerably impact freshwater ecosystems^17,18^.

Heatwaves below the water surface, particularly within lake sediments, remain poorly understood despite their potential to disrupt biogeochemical processes, as suggested in other aquatic systems^19^. Lake sediments are vital to the ecological functioning of lakes, serving as reservoirs for organic matter, nutrients and pollutants^20,21^. Their thermal regime governs key biogeochemical processes, including decomposition, nutrient cycling and greenhouse gas emissions, which are critical for maintaining ecosystem health^22–24^. Sediment-dwelling organisms, such as benthic invertebrates and microbial communities, are sensitive to temperature changes, which can alter their metabolic rates and reproductive success^25^. Moreover, elevated sediment temperatures, such as during sediment heatwaves, could accelerate the mineralization of organic matter, increasing the release of nutrients such as phosphorus and nitrogen into the water column^26^. This process can fuel harmful algal blooms, reduce oxygen levels and exacerbate eutrophication, further stressing aquatic ecosystems. Additionally, extreme sediment temperatures may alter the balance of redox-sensitive processes, such as denitrification and methanogenesis, with implications for greenhouse gas emissions^27,28^.

Understanding sediment heatwaves is essential for anticipating ecosystem responses to climate change. However, the duration and spatial extent of lake sediment heatwaves, both historically and in the future, remain unknown. This gap persists largely because lake sediment heatwaves cannot be resolved by satellite observations, and long-term, high-frequency in situ measurements of sediment temperatures are sparse and geographically limited^29–31^. As a result, sediment thermal extremes have remained largely inaccessible to observation-based analyses, necessitating a modelling-based approach to quantify their characteristics and responses to climate change. Moreover, previous global lake modelling studies have largely focused on surface water temperature, ice phenology and mixing dynamics, with sediment temperatures often omitted from analysis. Even when sediment thermal states are simulated, they have rarely been archived at sufficient temporal resolution or examined using extreme-event frameworks, and no standardized methodology has existed to define or compare sediment heatwaves across lakes or climate regimes. Together, these observational constraints and modelling limitations have prevented a systematic, global assessment of lake sediment heatwaves.

In this study, we address these knowledge gaps by applying a global, process-based lake modelling framework to provide a systematic assessment of the characteristics, historical trends and future evolution of lake sediment heatwaves under climate change. Specifically, we focus on sediment temperatures in both littoral and pelagic zones^32^. The littoral zone, comprising the nearshore areas of a lake, supports diverse biological activity and is particularly sensitive to surface temperature changes due to its proximity to the water surface. In contrast, the pelagic zone encompasses the deeper, open-water regions of a lake where sediments are less directly influenced by surface temperature fluctuations. Littoral sediments are heated by direct insolation and via heat transfer from the atmosphere through overlying, well-mixed surface waters. Here sediment temperatures often respond directly to atmospheric forcing and closely to near-surface water temperatures^33^. In contrast, pelagic sediments as defined in our study are often situated well below the photic zone and beneath the seasonal thermocline across which the surface heat flux is generally negligible^34^. Pelagic sediment temperatures are often related to hypolimnetic temperatures, but with a lower seasonal amplitude and a time lag^33,35^. As the thermocline erodes due to wind mixing or cooling, warm surface water is mixed down, and a heatwave signal could reach pelagic sediments. By examining lake sediment heatwaves in these distinct zones and their relationship with lake surface heatwaves, we aim to understand how they respond to climate change. To achieve this, we use a process-based lake model, driven by outputs from five General Circulation Models, under three Shared Socioeconomic Pathways (SSPs: SSP 1–2.6, SSP 3–7.0 and SSP 5–8.5) to simulate 41,499 representative lakes worldwide. This multi-model approach enables a robust analysis of historical (1981–2010) and future (2071–2100) changes in lake sediment heatwaves.

## Lake sediment heatwaves during the historic period

**Extended Data Fig. 1:**
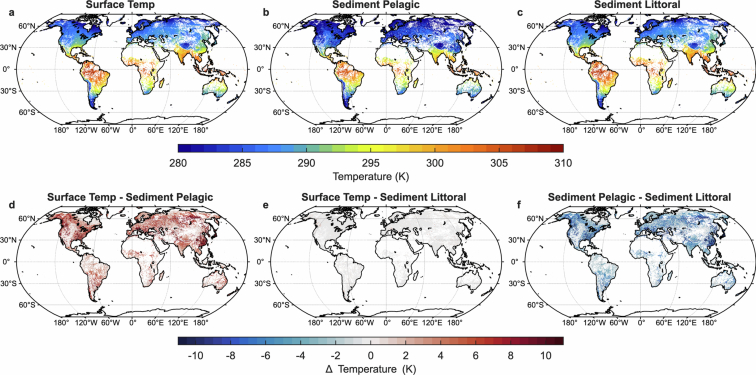
Historic lake surface and sediment temperature patterns. Shown are the average historic (1981-2010) temperatures of (**a**) lake surface waters, and the upper layer of lake sediments within (**b**) the pelagic, and **c**) the littoral zones. We also show the difference between (**d**) surface water temperature and sediment pelagic temperature, (**e**) surface water temperature and sediment littoral temperature, and (**f**) sediment pelagic and sediment littoral temperature. Basemaps generated with M_Map^51,52^.

**Extended Data Fig. 2:**
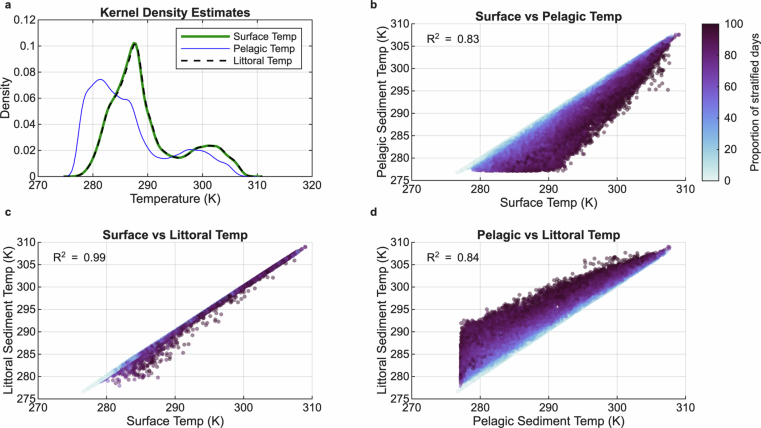
Lake temperature relationships and stratification effects. (**a**) Comparison of lake surface water temperature, littoral sediment temperature, and pelagic sediment temperature across the studied lakes. (**b**–**d**) Relationship between surface water temperature, pelagic sediment temperature, and littoral sediment temperature, and the proportion of annual stratified days in the studied lakes.

The thermal dynamics of lake sediments followed distinct spatial and temporal patterns, primarily reflecting larger scale climatic signals (Extended Data Fig. 1). Littoral sediment temperatures closely followed surface water temperatures whereas pelagic sediment temperatures showed a strong correlation with bottom water temperatures (Supplementary Fig. 1). On average, pelagic sediment temperatures were 3.3 K colder than littoral sediment temperatures (Extended Data Fig. 2). In some lakes, the difference between pelagic and littoral sediment temperatures could be much higher, with the largest difference calculated at 14.3 K. In the pelagic zone, the relationship between surface water temperature and lake sediment temperatures was influenced by the duration of thermal stratification, notably the proportion of the ice-free season that was stratified (Extended Data Fig. 2). Lakes with longer stratified periods, particularly those where stratification persisted for more than half of the ice-free season, exhibited greater differences between surface water temperature and pelagic sediment temperatures. The differences between littoral and pelagic sediment temperatures were greater in lakes that experienced a higher proportion of stratified days (Extended Data Fig. 2).

**Fig. 1:**
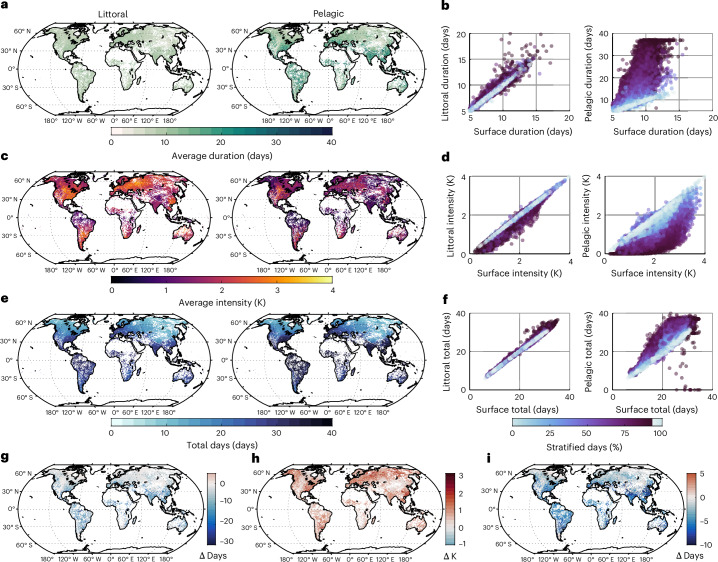
Littoral and pelagic lake sediment heatwaves. Key characteristics of littoral and pelagic sediment heatwaves during the historic period and their relationship with surface heatwave conditions. **a**, The average duration of sediment heatwaves in the littoral (left) and pelagic (right) zones. **b**, Compares the duration of littoral and pelagic sediment heatwaves with the duration of lake surface heatwaves and the proportion of stratified days. **c**,**d**, Similar to **a** and **b** but for the average intensity of heatwaves. **e**,**f**, Similar to **a** and **b** but for the total number of heatwave days per year. **g**, The difference in the average duration of sediment heatwaves between the littoral and pelagic zones (littoral minus pelagic; days). Negative values indicate longer-duration heatwaves in the pelagic zone relative to the littoral zone. **h**, The difference in the average intensity of sediment heatwaves between the littoral and pelagic zones (littoral minus pelagic; K). Positive values indicate more intense heatwaves in the pelagic zone relative to the littoral zone. **i**, The difference in the total number of sediment heatwave days per year between the littoral and pelagic zones (littoral minus pelagic; days). Negative values indicate a greater total number of heatwave days in the pelagic zone relative to the littoral zone. Basemaps in **a**,**c**,**e**,**g**–**i** generated with M_Map^51,52^.

**Extended Data Fig. 3:**
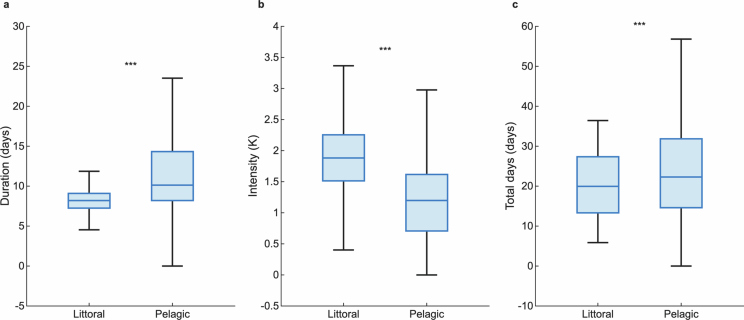
Differences between littoral and pelagic sediment heatwaves. Shown are differences between littoral and pelagic sediment heatwave metrics, including (**a**) duration, (**b**) intensity, and (**c**) total heatwave days per year. Boxplots display the median (center line), interquartile range (IQR; box limits), and whiskers extending to 1.5×IQR from the lower and upper quartiles. Outliers are not shown. Statistical differences between groups were assessed using Wilcoxon rank-sum tests. Asterisks indicate significance levels (*p < 0.05, **p < 0.01, ***p < 0.001). Sample sizes were identical across groups (n = 37,556 per habitat).

Using the daily simulated lake sediment temperatures for the littoral and pelagic zones, we defined and quantified several key characteristics of lake sediment heatwaves during the historic period (Fig. 1 and Extended Data Fig. 3). On average, the duration of lake sediment heatwaves in the littoral zone (hereafter referred to as littoral sediment heatwaves) was 8.2 ± 1.4 days (the uncertainty denotes the standard deviation), with an intensity of 1.9 ± 0.6 K and a total number of annual heatwave days of 20.3 ± 7.4 days. In contrast, lake sediment heatwaves in the pelagic zone (hereafter referred to as pelagic sediment heatwaves) were, on average, longer lasting with an average duration of 12.3 ± 6.5 days, although they were less intense, with an average intensity of 1.2 ± 0.6 K. Pelagic sediment heatwaves also accounted for more total heatwave days annually, reaching 23.0 ± 9.3 days per year. The differences in these sediment heatwave characteristics between littoral and pelagic zones also varied across regions (Fig. 1).

**Extended Data Fig. 4:**
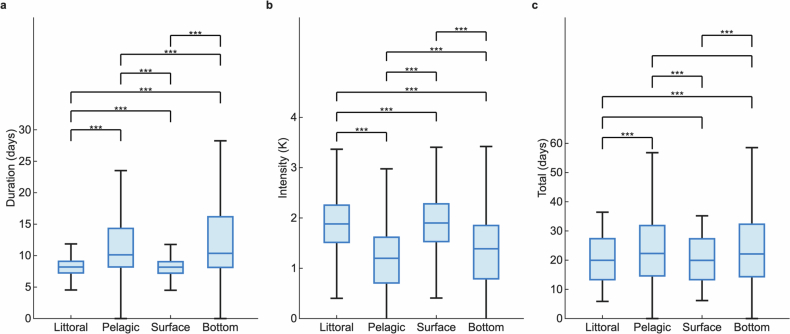
Comparison of heatwave characteristics across lake habitats. Shown are comparisons of heatwave characteristics across littoral, pelagic, surface, and bottom habitats, including (**a**) duration, (**b**) intensity, and (**c**) total heatwave days. Boxplots display the median (central line), interquartile range (IQR; box limits), and whiskers extending to 1.5×IQR from the first and third quartiles. Outliers are not shown. Statistical differences between groups were assessed using two-sided Wilcoxon rank-sum tests (Mann–Whitney U tests) for all pairwise comparisons among habitats. No correction for multiple comparisons was applied. Asterisks indicate significance levels (*p < 0.05, **p < 0.01, ***p < 0.001). Sample sizes were identical across groups (n = 37,556 per habitat).

The relationship between lake sediment and surface heatwaves, specifically in terms of their average intensity and duration, were strongest in the littoral zone (Supplementary Fig. 2). The intensity and duration of littoral sediment heatwaves increased almost linearly (slope = 0.98 and 0.95, respectively) with an increase in lake surface heatwaves. Thus, when surface water temperature observations are available, they could be used as a proxy for estimating the characteristics of littoral sediment heatwaves. For the pelagic zone, the relationship between the intensity and duration of lake sediment heatwaves with those of lake surface heatwaves were much more sporadic (slope = 0.6 and 0.1, respectively) and influenced by the proportion of stratified days during the open-water season (Supplementary Fig. 2). However, in lakes where the proportion of stratified days was short (for example, <10% of the ice-free season), the relationship between the intensity and duration of surface and pelagic sediment heatwaves were much stronger (slope = 1 and 0.94, respectively). This suggests that in mixed systems, surface heatwave characteristics could be used as a proxy for pelagic sediment heatwaves. However, pelagic sediment heatwave characteristics more closely match those of bottom heatwaves (mean duration = 13.4 ± 7.9 days; intensity = 1.3 ± 0.7 K; total count = 23.0 ± 9.7 days; Extended Data Fig. 4), which are defined relative to bottom water temperatures^36^. Pelagic sediment (and bottom) heatwaves are less intense, but longer lasting, than littoral (and surface) heatwaves.

**Extended Data Fig. 5:**
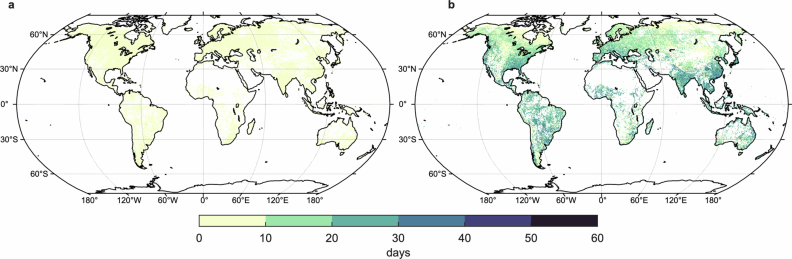
Sediment heatwaves occurring independently of surface heatwaves. (**a**) Number of days in which a littoral lake sediment heatwave occurs without a lake surface heatwaves. (**b**) Number of days in which a pelagic lake sediment heatwave occurs without a lake surface heatwaves. The results are shown for the historic period (1981-2010). Basemaps generated with M_Map^51,52^.

**Extended Data Fig. 6:**
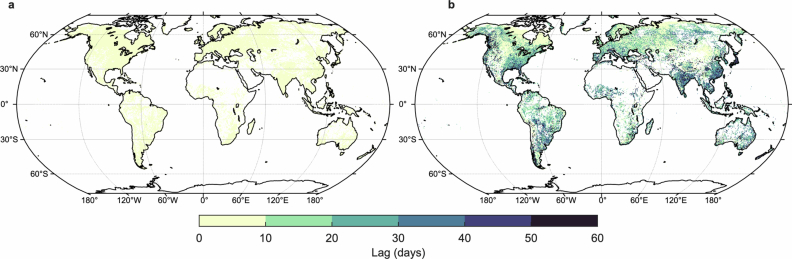
Timing offsets between sediment and surface heatwaves. (**a**) The lag (number of days) between the occurrence of a littoral lake sediment heatwave and lake surface heatwaves. (**b**) The lag between the occurrence of a pelagic lake sediment heatwave and lake surface heatwaves. The results are shown for the historic period (1981-2010). Basemaps generated with M_Map^51,52^.

A notable feature of pelagic sediment heatwaves was their occurrence independent of surface heatwave conditions (Extended Data Fig. 5). During the historic period, the average lag time between the occurrence of surface heatwaves and pelagic sediment heatwaves was 19.9 days, whereas for littoral sediment heatwaves this lag was less than 1 day (0.6 days, on average). On average, pelagic sediment heatwaves occurred for 14.9 days without a corresponding surface heatwave, while littoral sediment heatwaves occurred mostly within the same day as lake surface heatwaves (Extended Data Fig. 5). This disconnect between surface and pelagic sediment heatwaves can be largely attributed to the thermal lag between the surface and deeper regions of lakes, where sediments at greater depths respond more slowly to atmospheric and surface thermal conditions (Extended Data Fig. 6). This delay further highlights the decoupled thermal behaviour of pelagic sediments, which can retain heat long after surface conditions return to normal. Because littoral sediment heatwaves largely reflect changes in lake surface heatwaves, which have been extensively studied, this study will now focus primarily on future changes in pelagic sediment heatwaves, with results for littoral sediment heatwaves provided in Supplementary Figs. 6–9.

## Future projections of lake sediment heatwaves

**Fig. 2:**
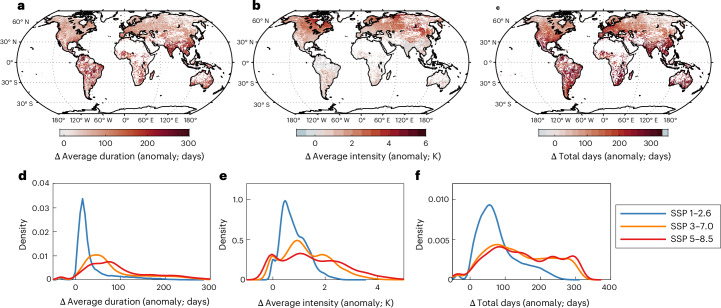
Pelagic lake sediment heatwaves under future climate change. **a**–**c**, Shown are changes in the average duration (**a**), average intensity (**b**) and total number of annual heatwave days (**c**) by the end of the twenty-first century (2071–2100) relative to the historic (1981–2010) period under SSP 5–8.5. **d**–**f**, The distribution of simulated changes in the average duration of pelagic sediment heatwaves (**d**), average intensity of pelagic sediment heatwaves (**e**) and total number of annual pelagic sediment heatwaves (**f**) under SSP 1–2.6, SSP 3–7.0 and SSP 5–8.5. Basemaps in **a**–**c** generated with M_Map^51,52^.

**Extended Data Fig. 7:**
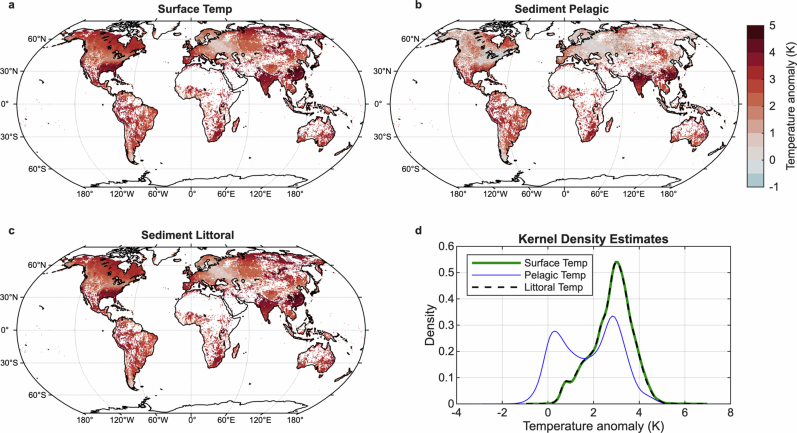
Projected changes in lake surface and sediment temperatures under SSP585. Future changes in (**a**) lake surface water temperature, (**b**) pelagic lake sediment temperature, and (**c**) littoral lake sediment temperature under SSP585. (**d**) Comparison of future changes in lake surface water temperature, littoral sediment temperature, and pelagic sediment temperature across the studied lakes. Basemaps in **a**–**c** generated with M_Map^51,52^.

Future projections of lake sediment heatwaves suggest an intensification in their duration, intensity and frequency (Fig. 2), largely reflecting climate-induced increases in their average temperatures (Supplementary Figs. 3 and 4 and Extended Data Fig. 7). These changes align with large-scale alterations in atmospheric forcing (Supplementary Fig. 5). Across the three SSPs (SSP 1–2.6, SSP 3–7.0 and SSP 5–8.5), substantial changes are anticipated, with the most severe effects expected under SSP 5–8.5, which represents the highest emissions scenario. Under SSP 1–2.6, the average duration of pelagic sediment heatwaves is projected to increase by 34.2 ± 40.5 days on average. The intensity of these heatwaves is also projected to rise, with temperature anomalies increasing by 0.8 ± 0.5 K relative to the historic period (that is, sediment heatwave intensity of 0.8 K higher than during the historic period). Additionally, the total number of heatwave days annually is expected to grow by 78.1 ± 57.2 days. Similarly, under SSP 3–7.0, the changes are more pronounced, with pelagic sediment heatwaves projected to last 80.0 ± 66.9 days longer, intensify by 1.3 ± 0.9 K and result in a 140.6 ± 89.4 increase in the total annual heatwave days. The most dramatic shifts occur under SSP 5–8.5, where pelagic sediment heatwaves are projected to become substantially more frequent and intense. The duration of pelagic sediment heatwaves is expected to increase by 96.0 ± 71.8 days, on average. Intensity increases of 1.5 ± 1.2 K above historical levels are projected, resulting in 149.7 ± 94.0 more annual heatwave days. Similar results for littoral sediment heatwaves are provided in Supplementary Fig. 6.

**Fig. 3:**
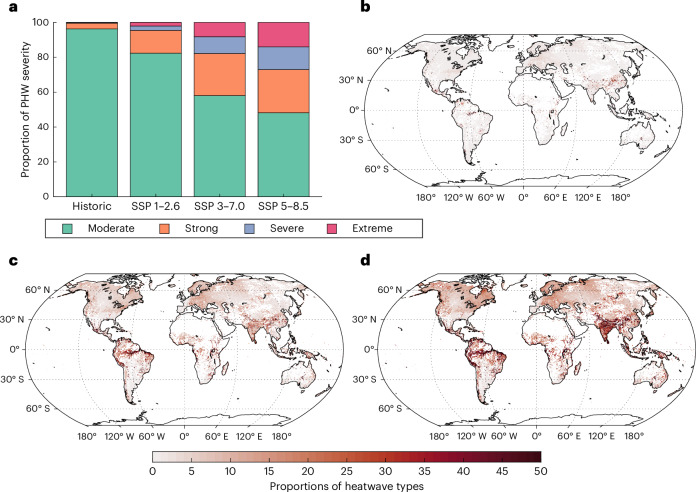
Severity of pelagic lake sediment heatwaves. **a**, The proportion of pelagic sediment heatwaves (PHW) globally that are categorized as moderate, strong, severe and extreme during the historic (1980–2010) and future (2071–2100) periods. Results for the latter are shown under SSP 1–2.6, SSP 3–7.0 and SSP 5–8.5. **b**–**d**, The spatial patterns in the proportion of strong (**b**), severe (**c**) and extreme (**d**) lake sediment heatwaves under SSP 5–8.5. Basemaps in **b**–**d** generated with M_Map^51,52^.

**Extended Data Fig. 8:**
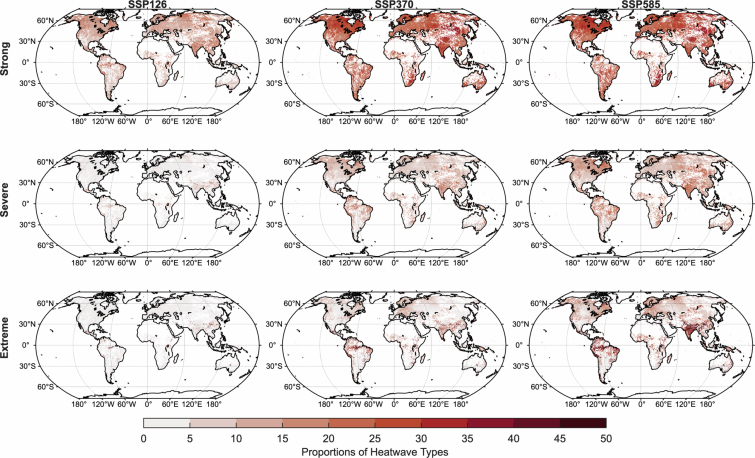
Future severity distribution of pelagic sediment heatwaves. Shown are the proportion of pelagic lake sediment heatwaves globally that are categorised as moderate, strong, severe, and extreme in the future (2071-2100). Results are shown under SSP126, SSP370, and SSP585. Basemaps generated with M_Map^51,52^.

In terms of severity, pelagic sediment heatwaves are projected to undergo a considerable shift this century. Most notably, under historic conditions, most (96%) pelagic sediment heatwaves were classified as moderate (Fig. 3), with only 3% categorized as strong and less than 1% as severe or extreme. However, future projections suggest an increase in the proportion of pelagic sediment heatwaves categorized as strong, severe or extreme, albeit with moderate heatwaves still often (except for SSP 5–8.5) being the most frequent category of heatwaves (Fig. 3). Under SSP 1–2.6, 82% of pelagic sediment heatwaves are projected as moderate, with 13% categorized as strong and the remainder (approximately 5%) as either severe or extreme. Under SSP 5–8.5, the proportion of pelagic sediment heatwaves classified as severe or extreme is projected to increase substantially by the end of the century, rising to 13% and 14% of all events, respectively. Additionally, less than half (48.2%) of pelagic sediment heatwave events will be categorized as moderate by 2071–2100 under SSP 5–8.5. An increase in the proportion of severe and extreme pelagic sediment heatwaves is especially notable in lakes situated at lower latitudes (Fig. 3c,d and Extended Data Fig. 8). Similar results for littoral lake sediment heatwaves are shown in Supplementary Figs. 7 and 8.

**Fig. 4:**
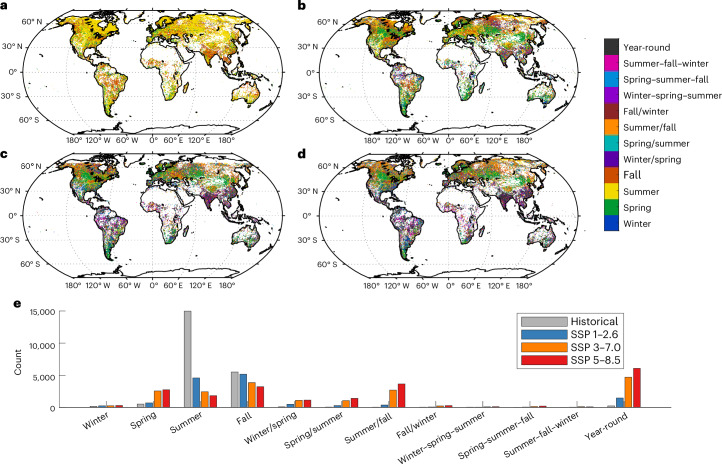
Seasonal occurrence of pelagic sediment heatwaves. **a**–**d**, The seasonality of pelagic sediment heatwaves during the historical period (**a**) and under future climate scenarios SSP 1–2.6 (**b**), SSP 3–7.0 (**c**) and SSP 5–8.5 (**d**). Shown are the season(s) in which pelagic sediment heatwaves most commonly occur. **e**, The dominant season (or multiple seasons) in which pelagic sediment heatwaves occur during the historic and future periods. We show the dominant season(s) during which sediment heatwaves took place. Basemaps in **a**–**d** generated with M_Map^51,52^.

Alongside the increased intensity of pelagic sediment heatwaves, the time of year in which they occur is also projected to change this century (Fig. 4 and Supplementary Fig. 9). During the historic period (Fig. 4a,e), most pelagic sediment heatwaves occurred during summer (69.2%) and fall (25.4%), with only a marginal proportion (2.2%) of lakes experiencing pelagic sediment heatwaves that extend beyond a single season (for example, 0.4% of lakes experienced pelagic sediment heatwaves that started in winter and ended in spring). This seasonal distribution is projected to change in the future, with multi-season pelagic sediment heatwaves becoming more common (Fig. 4). These changes are amplified by projected lengthening of the ice-free season (Supplementary Fig. 10), which varies globally and increases the period during which sediments are exposed to warming. Under SSP 1–2.6, multi-season pelagic sediment heatwaves are projected to increase by 12.7%, with some events extending across spring, summer and fall. However, the most notable shifts occur under SSP 5–8.5, where 59.8% of lakes are expected to experience pelagic sediment heatwaves that extend across multiple seasons by 2071–2100. Pelagic sediment heatwaves are expected to last year-round in 28.1% of lakes by 2071–2100 under SSP 5–8.5; this phenomenon will become increasingly prevalent in the tropics.

## Implications for the rate of biochemical processes

To estimate the potential impact of lake sediment heatwaves on the rate of biochemical processes in sediments, we computed fractional increases in relative sediment methanogenesis and respiration rates using an Arrhenius-type temperature sensitivity equation (equation (1) estimates dimensionless rate multipliers; Methods)^28,37,38^. In the littoral zone, from where most CH_4_ is typically emitted^39,40^, methane production more than doubled during future sediment heatwaves, increasing by a factor of 2.06, 2.22 and 2.28 under SSP 1–2.6, 3–7.0 and 5–8.5, respectively (Extended Data Table 1). Considering that heatwaves affect a portion of the year (equation (2) and Methods), we estimated the total increase in methane production at a factor of 1.47, 1.92 and 2.01, respectively (Extended Data Table 2). Pelagic sediment respiration, an often-dominant driver of seasonal oxygen depletion in lake bottom waters^41^, increased by a factor of 1.47, 1.52 and 1.53 under SSP 1–2.6, 3–7.0 and 5–8.5, respectively (Extended Data Table 1). Considering heatwave duration, total sediment respiration increased by a factor 1.26, 1.41 and 1.41, respectively (Extended Data Table 2).

## Discussion

This study provides a global-scale analysis of lake sediment heatwaves, offering insights into their historical patterns and future projections. Our results highlight several aspects: (1) littoral sediment heatwaves are closely linked to lake surface heatwaves; (2) pelagic sediment heatwaves exhibit a notable lag in response to extreme lake surface conditions, driven primarily by stratification duration; (3) pelagic sediment heatwaves are generally longer lasting and more frequent than littoral sediment heatwaves, with future projections suggesting an intensification of both; (4) the severity of pelagic sediment heatwaves will increase this century; (5) seasonality of pelagic sediment heatwaves will shift, with multi-seasonal or year-round events expected under SSP 5–8.5.

An important consequence of increasing sediment heatwaves is the enhanced release of methane, a potent greenhouse gas, alongside accelerated sediment respiration and organic carbon mineralization. Warming of lake sediments, particularly in the littoral zone, strongly influences methanogenesis, due to the temperature sensitivity of microbial communities and the exponential response of methane production to short-term thermal extremes^38,42^. Reduced oxygen penetration at higher temperatures and increased primary production in warmer climates further amplify substrate availability and methane fluxes^43–45^. Consistent with these processes, our model results indicate a doubling of total methane production in littoral sediments under SSP 5–8.5, which is probably to translate directly into higher emissions to the atmosphere via ebullition, the dominant pathway in productive lakes^46^. This estimate exceeds previous global projections of a 40% increase under severe warming^28^, in part because our analysis distinguishes between littoral and pelagic zones. Pelagic sediment respiration is also projected to increase by 26–41%, potentially exacerbating oxygen depletion in bottom waters^47,48^. These impacts may be further intensified by interactions with eutrophication, which can enhance both methane emissions and oxygen depletion^49,50^. Whereas methane and carbon dioxide emissions are not explicitly quantified here, our results highlight the critical role of sediment heatwaves in amplifying biogeochemical fluxes beyond what would be expected from mean warming alone, emphasizing the need for further research on their contribution to greenhouse gas emissions and the carbon balance of lakes.

The timing and seasonality of sediment heatwaves are projected to shift under future warming, with likely ecological implications. Under SSP 5–8.5, many lakes may experience year-round heatwaves and multi-seasonal events are expected to become increasingly common by the end of the century. Such prolonged thermal stress could limit the ability of benthic organisms and other aquatic life to recover, contrasting with historical conditions where most heatwaves occurred within a single season. Lakes at lower latitudes are likely to experience the most severe impacts, where high temperatures will compound additional stressors such as nutrient pollution, water scarcity and habitat degradation, potentially complicating adaptation and management strategies.

Our study represents a global assessment of lake sediment heatwaves, providing valuable insights into their future trajectories under climate change. The projected intensification of these events highlights the need for comprehensive management strategies that consider both surface and subsurface thermal conditions. Changing thermal regimes increasingly threaten the ecological integrity of lakes. Proactive conservation and management will therefore be essential in a warming world, including subsurface temperature monitoring, protection of cold-water and sediment refugia and reduction of external stressors that amplify thermal extremes.

## Methods

### Simulations of lake water and sediment temperatures

Lake temperatures investigated in this study were simulated, within the ISIMIP (Inter-Sectoral Impact Model Intercomparison Project) phase 3b (ISIMIP3b) lake sector, with the Advanced Lake Biogeochemistry Model (ALBM)^53–55^. Among the lake models contributing to ISIMIP3b, ALBM was selected because it explicitly simulates vertically resolved sediment temperatures, which are required to identify and quantify sediment heatwaves; other models in ISIMIP3b focus on water column thermal dynamics. Following the ISIMIP3b global lake sector protocol, ALBM was used to simulate lake water and sediment temperatures at a 0.5°-by-0.5° grid resolution that are represented by 41,449 grid-cell-specific representative lakes. For each grid cell, a single representative lake is defined, whose surface area corresponds to the aggregated area of all lakes within that grid cell and whose bathymetry is derived from global depth–area relationships scaled to that representative lake (Supplementary Fig. 11). The grid-scale fractions of lakes within each grid were determined by HydroLakes^56^ and the depth–area hypsographic curves of representative lakes were extracted from GLOBathy^57^. To drive ALBM, bias-corrected General Circulation Model (GCM) projections from ISIMIP3b were used, specifically projections from GFDL-ESM4, IPSL-CM6A-LR, MPI-ESM1-2-HR, MRI-ESM2-0 and UKESM1-0-LL for historic (1901–2014) and future (2015–2100) periods under three SSPs (SSP 1–2.6, SSP 3–7.0, SSP 5–8.5). The variables used to drive ALBM included daily air temperature, wind speed, surface downwelling solar and thermal radiation, total and snow precipitation and relative humidity.

For each representative lake, ALBM was configured with a single one-dimensional water column shaped according to the lake’s hypsographic curve, and four laterally aggregated sediment columns, each discretized into 40 vertical layers. The water column was divided into 50 layers of variable thickness, increasing with depth to resolve near-surface thermal gradients and deeper stratification; a schematic diagram of this component of the model set-up is provided in Fig. S1 of ref. ^55^. The use of four sediment columns represents a compromise between spatial realism and computational efficiency for global simulations. The sediment columns correspond to different depth zones of the lake, allowing lateral heterogeneity in sediment–water heat exchange to be represented without resolving full horizontal dynamics. Specifically, the first sediment column underlies the shallowest water layers, extending from the surface to the depth at which the cumulative lake area equals 75% of the total surface area, and is defined here as littoral sediment. The fourth sediment column underlies the deepest water layers, extending from the depth at which the cumulative area equals 25% of the surface area to the lake bottom, and is defined as pelagic sediment. The intermediate columns represent transitional depth zones and are not analysed explicitly in this study.

ALBM uses variable layer thickness to represent water and sediment columns. Specifically, the layer thickness of water and sediment columns increases exponentially from the surface to the bottom. The thickness of the first water and sediment layers is always roughly 0.1 m. The first 1 m water and sediment depths are usually represented by at least five layers. This discretization scheme ensures that the water and sediment layers where intense thermal dynamics occur can be resolved properly. As a result, the applied discretization can achieve the resolution needed for the analysis and validation at 0.5-m or 1-m sediment depths

The initial temperature of the sediment columns was set to change linearly from 4 °C to a lake-specific reference temperature at the sediment bottom, derived from the annual mean air temperature^29^. To produce reasonable initial conditions for historic simulations, ALBM was first spun up for 50 years using the GCM data from 1850 repetitively and then run using prognostic GCM data from 1850 to 1901. In this study, we investigate sediment temperatures (littoral and pelagic) located at a depth of 1 m below the surface sediment.

### Validation

ALBM has been tested extensively in past studies including detailed validations across a spectrum of lake contexts. For example, ALBM has previously been used to simulate lake temperature and changes to stratification and mixing in lakes and has been shown to accurately reproduce temporal variations in bottom water^58,59^ and sediment temperatures^60^. In a study of 58 lakes, the ALBM-simulated temperatures of hypolimnetic waters achieved a mean error of less than 1.2 °C and a correlation coefficient close to 0.95 (ref. ^61^). Also, a multi-model comparison study indicated that ALBM’s modelling skill of water temperature does not deteriorate with depth for deep lakes^62^.

Given the limited availability of direct sediment temperature measurements, this study utilized bottom water temperature as a proxy for validating sediment surface temperatures. The strong relationship between lake bottom water temperature and sediment surface temperature, as illustrated in Supplementary Fig. 1, justifies this approach. To further ensure the reliability of ALBM outputs for this study, model simulations of lake sediment temperatures were systematically compared with observational bottom water temperature data from lakes spanning a range of latitudes, climatic conditions and morphometric features.

The observational dataset used for validation was compiled from three key sources^48,63,64^, providing vertical profiles of water temperature. Observations were matched to individual lakes using HYDROLakes v1.0 polygons, ensuring spatial accuracy by retaining only lakes where the HYDROLake-estimated surface area deviated by no more than 30% from the surface area reported in the observational datasets. The deepest measurement in temperature profiles extending down to >70% of the lake’s maximum depth (derived from bathymetric maps or direct measurements) are considered as lake bottom temperature. Model validation is performed by comparing these deepest measurements with the simulation from ALBM. We only include lake bottom temperatures measured at depths comparable with the maximum depth applied in the ISIMIP3b simulations, with absolute relative difference smaller than 0.3. In total, we have 10,485 lake bottom temperature measurements from 138 lakes during the historical period (Supplementary Fig. 12). Good comparison is observed between the in situ measurements and ALBM, validating ALBM’s capacity to simulate bottom water temperatures, thereby reinforcing the reliability of its sediment temperature predictions.

Observations of lake sediment temperatures are rare^29–31^. However, we identified some published datasets in the literature, including in situ measurements from six lakes (Wingra, Ryan, Taihu, Mendota, Inre Harrsjön and Mellersta Harrsjön; Supplementary Fig. 12), which we use here as an additional form of validation. Lake Wingra is a small (130 ha) and shallow (maximum depth = 4.3 m) lake located in Wisconsin, USA. The measurement was conducted on the lake sediment surface in the pelagic zone from June to August, 2006 (refs. ^65,66^). Lake Ryan is another small (6.1 ha) lake with maximum depth close to 11 m located in Minnesota (USA). Data were collected 0.5 m below the sediment surface in the pelagic zone from November (1989) to April (1990) reported by ref. ^66^. Lake Mendota is also located in Wisconsin (USA) but much larger (3,940 ha) and deeper (maximum depth = 25.3 m) than Lake Wingra. Lake sediment temperatures were measured on the sediment surface both in littoral (depth of 8 m) and pelagic (depth of 23.5 m) zones from 1918 to 1920 (ref. ^33^). Lake Taihu is the third-largest freshwater lake in China (225,000 ha) with a maximum depth of 3~4 m. We compiled sediment temperature data of 2016 from 14 sites, including 12 (littoral) nearshore locations across the eastern, southern, western and northern zones and two (pelagic) in the central region^67^. Inre and Mellarsta Harrsjön are two small lakes in northern Sweden with surface areas of 2.2 and 1.1 ha and maximum depths of 5 m and 6.7 m, respectively. Temperature moorings were deployed at the lakes’ deepest points from 2009 to 2019, with the bottom temperature sensor buried in the soft gyttja sediment with the mooring anchor at 5–10 cm depth^31^. We compared these data with the ALBM simulations up to 2015, aligning with the historical period defined in ISIMIP3b. When comparing the in situ measurements and the ALBM simulations for each lake, we chose the nearest ISIMIP3b grid with comparable maximum lake depth. We calculated good comparisons between ALBM and field measurements in these lakes (small, large, shallow and deep).

Previous validation of ALBM against sediment temperature observations has also been conducted for Goldstream Lake, Alaska^51^. Modelled temperatures were compared with measurements at both the lake centre and margin, including the water–sediment interface and sediment layers up to 1 m depth. The model reproduced observed thermal dynamics with mean errors generally below 0.6 °C in the water column and below 0.3 °C in sediments.

### Lake heatwaves

Lake heatwaves were quantified following the approach of ref. ^8^. Heatwave occurrence, average duration, average intensity and the annual number of heatwave days were identified using the R package heatwaveR^68^. For each lake and depth zone, heatwaves were defined as periods of at least five consecutive days during which daily temperatures exceeded a seasonally varying 90th-percentile threshold derived from a fixed historical reference period (1981–2010). The daily climatological mean and percentile thresholds were calculated using an 11-day moving window centred on each calendar day and smoothed with a 31-day moving average. Two events separated by fewer than 2 days were merged into a single heatwave. Heatwave intensity was defined as the mean temperature anomaly relative to the seasonal climatology during each event, and duration was calculated as the time between event onset and termination. Heatwave metrics were calculated for the ice-free season. The historical climatology and thresholds were applied consistently to both historical and future simulations, enabling assessment of changes in heatwave characteristics relative to present-day thermal conditions. Surface, bottom and sediment heatwaves were identified independently based on their respective temperature time series, with sediment heatwaves defined separately for littoral and pelagic sediments. Although the primary focus of our analysis was on annual (ice-free) heatwave metrics, we also conducted seasonal comparisons (January–March, April–June, July–September and October–December) to examine the frequency of occurrence of lake surface and sediment heatwaves. For this study, June was used as the start of boreal summer and December as the start of austral summer. When calculating the time series of annual average intensity and duration of lake heatwave events, we separated heatwaves into distinct events if they extended beyond 31 December, thereby allowing for a maximum heatwave duration of 366 days.

To classify the strength of lake heatwaves, we used an intensity-based categorization. Each event was classified as Moderate, Strong, Severe or Extreme, based on the maximum intensity relative to a threshold temperature anomaly exceeding the climatological mean. Specifically: (1) moderate events exceeded the threshold but were less than twice the threshold anomaly; (2) strong events exceeded twice the threshold but were less than three times the threshold anomaly; (3) severe events exceeded three times the threshold and (4) extreme events exceeded four times the threshold anomaly.

We calculated the time of year in which lake sediment heatwaves most frequently occur during the historic and future periods. During each period, we defined the season(s) during which a heatwave occurred and then calculated the most frequent seasonal occurrence. The season of occurrence could either be a single season (for example, summer) or they could extend across two (for example, winter/spring) or three (for example, winter–summer) seasons. Events lasting across four or more seasons are defined as year-round.

These lake heatwave metrics were calculated using ALBM driven by five GCMs. The results presented represent the average across all individual GCM outputs.

### Stratification and mixing

We defined a lake as stratified when the temperature difference between the surface and bottom water exceeded 1 °C (refs. ^69–71^). The proportion of stratified days was calculated as the ratio of stratified days to the total annual ice-free days. Ice cover was defined as when lake surface water temperatures were 0 °C.

### Cross-correlation and temporal synchrony

To assess temporal synchrony between lake surface and sediment thermal conditions, we quantified the lag between surface water and sediment temperatures using cross-correlation analysis. For each lake, Pearson’s correlation coefficients between daily bottom water temperature and daily sediment temperature were first calculated using the *cor* function in R. To estimate temporal lags, we computed the cross-correlation function between paired daily water and sediment temperature time series using the *ccf* function in R^72^.

### Heatwave impacts on biochemical processes

We used an Arrhenius-type exponential temperature function^38^ characterizing the overall temperature sensitivity of methanogenesis and respiration to project rates in lake sediments under historic and future sediment heatwaves:

1 $$J\left(T\right)=J\left({T}_{{\rm{c}}}\right){e}^{{{E}_{{\rm{a}}}}^{{\prime} }\left(\frac{1}{{k}_{{\rm{B}}}{T}_{{\rm{c}}}}-\frac{1}{{k}_{{\rm{B}}}T}\right)}$$

where *J* is the methanogenesis or respiration rate, *T* is the absolute temperature in Kelvin (K), *E*_a_′ is the empirical activation energy in electron volts (eV) and *k*_B_ is the Boltzmann constant (8.62 × 10^−5^ eV K^−1^). *E*_a_′ is the temperature sensitivity of the rate and is equivalent to the slope of the centred rate ($\mathrm{ln}(J\left(T\right))-\mathrm{ln}(J\left({T}_{{\rm{c}}}\right)$)) and temperature ($1/{k}_{{\rm{B}}}{T}_{{\rm{c}}}-1/{k}_{{\rm{B}}}T$), where *J*(*T*_c_) is the rate at a midpoint temperature *T*_c_. We used robust *E*_a_′ values for methanogenesis (*E*_a_′ = 0.96 eV) (ref. ^38^) and organic carbon mineralization (*E*_a_′ = 0.65 eV) (ref. ^37^) from global syntheses of lake sediment incubation measurements. *T* is the sum of the historic ice-free mean sediment temperature and the mean heatwave intensity in each 0.5° grid cell. *T*_c_ is the global historic ice-free mean sediment temperature derived from model output, 289.95 K and 286.55 K in the littoral and pelagic zone, respectively. Using equation (1), we computed rates (*J*(*T*)) as departures from an arbitrary historical global mean rate (*J*(*T*_c_) = 1)^28^. By computing dimensionless rates, we obtained relative, direct temperature effects and omitted the need for biological modulating variables required to model absolute rates (for example, lake trophic state or sediment organic carbon content)^55^, which are not yet available as a global gridded dataset. In other words, *J(T)* represents the fractional change in methanogenesis and respiration rates as a function of the departure from *T*_c_.

The change in annual mean relative rates also factors in the duration of heatwaves.

2 $${J}_{\mathrm{tot}}=\frac{{t}_{\mathrm{hw},\mathrm{tot}}}{365}\left(J\left(T\right)-1\right)+1$$

where *J*_tot_ is the fractional change in annual mean methanogenesis or respiration rates, assuming a rate of 1 during non-heatwave days, and *t*_hw,tot_ is the total annual number of heatwave days. Because *T* in *J(T)* reflects the mean temperature during heatwave periods only rather than the mean annual temperature, heatwave intensity and duration are not double-counted in equation (2). The assumption of a rate of 1 outside the heatwave periods further ensures that we only assess the direct impact of heatwaves rather than the broader impact of climate warming. Our estimates of *J(T)* and *J*_tot_ are probably conservative because the use of long-term mean temperatures omits temperature peaks during heatwaves which would have a disproportionate impact on methanogenesis and respiration due to the nonlinear nature of the Arrhenius-type temperature relation (equation (1)).

We assumed here that the *E*_a_′ values (equation (1)) will be constant over time, while laboratory and mesocosm experiments suggest *E*_a_′ might change due to synergistic temperature effects, such as adaptation of microbial communities, enhanced primary production or eutrophication^49^. However, the wide variety of natural aquatic ecosystems sampled to obtain the mean *E*_a_′ values in ref. ^37^ and ref. ^38^ have widely different properties and warming histories yet still converge to very similar temperature relations. In a sensitivity analysis conducted as part a global lake methanogenesis study, *E*_a_′ values were changed randomly in each grid cell within a qualified uncertainty range, and global patterns in *J(T)* did not change considerably^28^. This suggests that *E*_a_′ values are robust over space and time within the expected warming range, though more research on this topic is clearly warranted.

### Further considerations

The interpretation of our results requires careful consideration of the modelling framework and associated uncertainties. Our simulations are based on a physically resolved representation of lake thermal dynamics, capturing vertical temperature gradients, stratification and sediment thermal properties. The projected intensification of sediment heatwaves reflects the combined influence of climate forcing trends, including rising air temperatures, increased solar radiation, changing wind speeds and extended ice-free seasons, which lengthen the period of potential thermal stress. Uncertainty arises from multiple sources: (1) parametrization of sediment thermal properties and heat fluxes within the modelling framework, (2) simplifications in vertical mixing and stratification processes and (3) variability in climate forcing from different GCMs and emissions scenarios. Additional limitations include the absence of horizontal heat advection along shorelines, temporally varying water levels and flooding or drying of littoral regions, which could influence sediment thermal dynamics but are not represented in the one-dimensional model structure. In terms of the impact of sediment heatwaves on biochemical processes, we also emphasize that our estimates intentionally isolate the effect of temperature and do not account for other controls on biogeochemical processes, such as sediment organic carbon availability, redox conditions, oxygen penetration depth or microbial community composition. As such, the results should be interpreted as indicative of potential sensitivity of sediments to extreme warming, rather than precise predictions of sediment methanogenesis and respiration rates.

An additional source of uncertainty arises from differences among the GCMs used to force ALBM. The five GCMs differ in their representation of regional air temperature trends, radiation balance, wind forcing and seasonal ice dynamics, which influence the magnitude and timing of simulated lake warming and consequently the intensity and duration of sediment heatwaves. These differences introduce spread in the projected rate and regional expression of change. However, across all climate forcings, the simulations consistently indicate increases in the frequency, duration and cumulative intensity of sediment heatwaves under continued warming. We therefore interpret inter-model variability primarily as uncertainty in magnitude rather than in the direction of change, with the emergence of more frequent and prolonged sediment heatwaves representing a robust response to projected climate warming.

A key methodological choice in our analysis was the use of a fixed historical baseline for defining sediment heatwaves. This baseline was applied consistently to both historical and future simulations, allowing extreme thermal conditions to be quantified relative to present-day climates. Under strong warming scenarios, this approach highlights the emergence of sediment temperatures that exceed historical heatwave thresholds for extended periods, including across multiple seasons or year-round in some regions. While shifting-baseline definitions examine variability around a warming mean state, they can mask the occurrence of absolute thermal extremes and decouple physical forcing from biological and biogeochemical responses. A fixed-baseline approach aligns with established practice in atmospheric and marine heatwave studies^10^, recommendations from the World Meteorological Organization and recent climate assessments, ensuring comparability across systems and facilitating evaluation of ecosystem exposure to absolute thermal stress.

## Online content

Any methods, additional references, Nature Portfolio reporting summaries, source data, extended data, supplementary information, acknowledgements, peer review information; details of author contributions and competing interests; and statements of data and code availability are available at 10.1038/s41561-026-01986-3.

## Supplementary information

Supplementary Information Supplementary Figs. 1–12.

Peer Review File

## Data Availability

The lake sediment heatwave simulations are available via Zenodo at 10.5281/zenodo.18507326 (ref. ^73^).

## Extended data

**Extended Data Table 1:** Global responses of lake sediment methanogenesis and respiration to sediment heatwaves

		Methanogenesis			Respiration		
Zone	Scenario	mean	95% CI	range	mean	95% CI	range
Littoral	Historic	1.89	1.87-1.91	0.16-11.74	1.4	1.39-1.41	0.29-5.30
	Future, SSP 126	2.06	2.04-2.08	0.18-12.21	1.5	1.49-1.51	0.31-5.44
	Future, SSP 370	2.22	2.20-2.24	0.20-12.19	1.59	1.58-1.60	0.33-5.44
	Future, SSP 585	2.28	2.26-2.30	0.21-12.21	1.63	1.62-1.64	0.34-5.44
Pelagic	Historic	1.92	1.90-1.95	0.26-15.19	1.39	1.37-1.40	0.40-6.31
	Future, SSP 126	2.09	2.06-2.11	0.27-16.04	1.47	1.46-1.48	0.41-6.55
	Future, SSP 370	2.16	2.14-2.18	0.27-16.56	1.52	1.51-1.53	0.41-6.69
	Future, SSP 585	2.18	2.15-2.20	0.27-16.12	1.53	1.52-1.55	0.41-6.57

Global mean fractional change in lake sediment methanogenesis and respiration rates during historic and future sediment heatwaves, relative to the rate at the historic ice-free mean sediment temperatures (Eq. 1). 95% confidence intervals are bias-corrected and accelerated (BCa) intervals computed using 5000 replicates.

**Extended Data Table 2:** Global impacts of sediment heatwaves on annual lake sediment methanogenesis and respiration

		Methanogenesis			Respiration		
Zone	Scenario	mean	95% CI	range	mean	95% CI	range
Littoral	Historic	1.08	1.07-1.08	0.93-1.97	1.04	1.04-1.04	0.94-1.39
	Future, SSP 126	1.47	1.46-1.48	0.40-8.29	1.23	1.22-1.23	0.51-4.05
	Future, SSP 370	1.92	1.90-1.94	0.29-10.43	1.45	1.44-1.45	0.42-4.86
	Future, SSP 585	2.01	1.99-2.03	0.26-10.41	1.5	1.49-1.50	0.39-4.81
Pelagic	Historic	1.09	1.09-1.09	0.72-1.63	1.04	1.04-1.04	0.72-1.63
	Future, SSP 126	1.57	1.56-1.59	0.51-11.91	1.26	1.26-1.27	0.62-5.10
	Future, SSP 370	1.87	1.85-1.89	0.39-15.05	1.41	1.40-1.41	0.51-6.19
	Future, SSP 585	1.86	1.85-1.88	0.37-14.69	1.41	1.40-1.41	0.50-6.04

Global mean fractional change in total annual lake sediment methanogenesis and respiration due to sediment heatwaves, that is considering the duration of heatwaves (Eq. 2). 95% confidence intervals are bias-corrected and accelerated (Bca) intervals computed using 5000 replicates.

## Code availability

The code for the analysis and figures is available via Zenodo at 10.5281/zenodo.18507326 (ref. ^73^).

## References

[1] Seneviratne, S. I. et al. in *Climate Change 2021: The Physical Science Basis* (eds Masson-Delmotte, V. et al.) 1513–1766 (Cambridge Univ. Press, 2021).

[2] Barriopedro, D. et al. Heat waves: physical understanding and scientific challenges. *Rev. Geophys.* **61**, e2022RG000780 (2023).

[3] Sun, D. et al. Frequent marine heatwaves hidden below the surface of the global ocean. *Nat. Geosci.* **16**, 1099–1104 (2023).

[4] Perkins-Kirkpatrick, S. E. & Lewis, S. C. Increasing trends in regional heatwaves. *Nat. Commun.* **11**, 3357 (2020). 32620857 10.1038/s41467-020-16970-7 PMC7334217

[5] Perkins-Kirkpatrick, S. E. Increasing frequency, intensity and duration of observed global heatwaves and warm spells. *Geophys. Res. Lett.* **39**, L20714 (2012).

[6] Domeisen, D. I. V. et al. Prediction and projection of heatwaves. *Nat. Rev. Earth Environ.* **4**, 36–50 (2023).

[7] Frölicher, T. L., Fischer, E. M. & Gruber, N. Marine heatwaves under global warming. *Nature* **560**, 360–364 (2018). 30111788 10.1038/s41586-018-0383-9

[8] Woolway, R. I. et al. Lake heatwaves under climate change. *Nature* **589**, 402–407 (2021). 33473224 10.1038/s41586-020-03119-1

[9] Tassone, S. J. et al. Increasing heatwave frequency in streams and rivers of the United States. *Limnol. Oceanogr. Lett.* **8**, 295–304 (2023).

[10] Hobday, A. J. et al. A hierarchical approach to defining marine heatwaves. *Prog. Oceanogr.* **141**, 227–238 (2016).

[11] Oliver, E. C. J. et al. Longer and more frequent marine heatwaves over the past century. *Nat. Commun.* **9**, 12 (2018). 29636482 10.1038/s41467-018-03732-9 PMC5893591

[12] Smale, D. A. et al. Marine heatwaves threaten global biodiversity and the provision of ecosystem services. *Nat. Clim. Change* **9**, 306–312 (2019).

[13] Smith, K. E. et al. Socioeconomic impacts of marine heatwaves: global issues and opportunities. *Science* **374**, eabj3593 (2021). 34672757 10.1126/science.abj3593

[14] Woolway, R. I., Albergel, C., Frölicher, T. L. & Perroud, M. Severe lake heatwaves attributable to human-induced global warming. *Geophys. Res. Lett.* **49**, e2021GL097031 (2022).

[15] Wang, X. et al. Climate change drives rapid warming and increasing heatwaves of lakes. *Sci. Bull.* **68**, 1574–1584 (2023). 10.1016/j.scib.2023.06.028 37429775

[16] Wang, X. et al. Disproportionate impact of atmospheric heat events on lake surface water temperature increases. *Nat. Clim. Change* **14**, 1172–1177 (2024).

[17] Tye, S. P. et al. Climate warming amplifies the frequency of fish mass mortality events across north temperate lakes. *Limnol. Oceanogr. Lett.* **7**, 510–519 (2022).

[18] Duan, H. et al. Warming surface and lake heatwaves as key drivers to harmful algal blooms: a case study of Lake Dianchi, China. *J. Hydrol.* **632**, 130970 (2024).

[19] Tassone, S. J. & Pace, M. L. Increased frequency of sediment heatwaves in a Virginia seagrass meadow. *Estuaries Coasts* **47**, 656–669 (2024).

[20] Sobek, S. et al. Organic carbon burial efficiency in lake sediments controlled by oxygen exposure time and sediment source. *Limnol. Oceanogr.* **54**, 2243–2254 (2009).

[21] Einsele, G., Yan, J. & Hinderer, M. Atmospheric carbon burial in modern lake basins and its significance for the global carbon budget. *Glob. Planet. Change* **30**, 167–195 (2001).

[22] Duc, N. T., Crill, P. & Bastviken, B. Implications of temperature and sediment characteristics on methane formation and oxidation in lake sediments. *Biogeochemistry* **100**, 185–196 (2010).

[23] Fuchs, A. et al. Effects of increasing temperatures on methane concentrations and methanogenesis during experimental incubation of sediments from oligotrophic and mesotrophic lakes. *J. Geophys. Res. Biogeosci.* **121**, 1394–1406 (2016).

[24] Weyhenmeyer, G. et al. Global lake health in the Anthropocene: societal implications and treatment strategies. *Earth’s Future* **12**, e2023EF004387 (2024).

[25] Palmer, M. A. et al. Linkages between aquatic sediment biota and life above sediments as potential drivers of biodiversity and ecological processes. *BioScience* **50**, 1062–1075 (2000).

[26] Søndergaard, M., Jensen, J. P. & Jeppesen, E. Role of sediment and internal loading of phosphorus in shallow lakes. *Hydrobiologia* **506**, 135–145 (2003).

[27] Davidson, E. A., Kanter, D. & Gurney, K. R. Methane emissions from freshwater ecosystems: implications for global methane budgets. *Glob. Change Biol.* **21**, 2813–2827 (2015).

[28] Jansen, J. et al. Global increase in methane production under future warming of lake bottom waters. *Glob. Change Biol.* **28**, 5427–5440 (2022). 10.1111/gcb.16298 PMC9546102 35694903

[29] Fang, X. & Stefan, H. G. Temperature variability in lake sediments. *Water Resour. Res.* **34**, 717–729 (1998).

[30] Cyr, H. Temperature variability in shallow littoral sediments of Lake Opeongo (Canada). *Freshwater Sci.* **31**, 895–907 (2012).

[31] Crill, P., Wik, M. & Jansen, J. Temperatures in subarctic lakes on the Stordalen Mire, Abisko, Northern Sweden. Dataset version 4. *Bolin Centre Database*10.17043/stordalen-lake-temperatures-4 (2021).

[32] Wetzel, R. G. *Limnology* 3rd edn (Elsevier, 2001).

[33] MacIntyre, S. & Hamilton, D. P. in *Wetzel’s Limnology* 95–153 (Elsevier, 2024).

[34] Nakhaei, N., Ackerman, J. D., Bouffard, D., Rao, Y. R. & Boegman, L. Empirical modeling of hypolimnion and sediment oxygen demand in temperate Canadian lakes. *Inland Waters* **11**, 351–367 (2021).

[35] Birge, E. A., Juday, C. & March, H. W. The temperature of the bottom deposits of Lake Mendota; a chapter in the heat exchanges of the lake. *Trans. Wis. Acad. Sci. Arts Lett.* **23**, 187e231 (1927).

[36] Woolway, R. I. et al. Subsurface heatwaves in lakes. *Nat. Clim. Change* **15**, 554–559 (2025). 10.1038/s41558-025-02314-0 PMC12064439 40353068

[37] Gudasz, C. et al. Temperature-controlled organic carbon mineralization in lake sediments. *Nature* **466**, 478–481 (2010). 20651689 10.1038/nature09186

[38] Yvon-Durocher, G. et al. Methane fluxes show consistent temperature dependence across microbial to ecosystem scales. *Nature* **507**, 488–491 (2014). 24670769 10.1038/nature13164

[39] Juutinen, S. et al. Major implication of the littoral zone for methane release from boreal lakes. *Glob. Biogeochem. Cycles* **17** (2003).

[40] Wik, M., Crill, P. M., Varner, R. K. & Bastviken, D. Multiyear measurements of ebullitive methane flux from three subarctic lakes. *J. Geophys. Res. Biogeosci.* **118**, 1307–1321 (2013).

[41] Livingstone, D. M. & Imboden, D. M. The prediction of hypolimnetic oxygen profiles: a plea for a deductive approach. *Can. J. Fish. Aquat. Sci.* **53**, 924–932 (1996).

[42] Tveit, A. T., Urich, T., Frenzel, P. & Svenning, M. M. Metabolic and trophic interactions modulate methane production by Arctic peat microbiota in response to warming. *Proc. Natl Acad. Sci. USA* **112**, E2507–E2516 (2015). 25918393 10.1073/pnas.1420797112 PMC4434766

[43] Sobek, S. et al. Temperature dependence of apparent respiratory quotients and oxygen penetration depth in contrasting Lake sediments. *J. Geophys. Res. Biogeosci.* **122**, 3076–3087 (2017).

[44] Yvon-Durocher, G., Hulatt, C. J., Woodward, G. & Trimmer, M. Long-term warming amplifies shifts in the carbon cycle of experimental ponds. *Nat. Clim. Change* **7**, 209–213 (2017).

[45] Grasset, C. et al. Large but variable methane production in anoxic freshwater sediment upon addition of allochthonous and autochthonous organic matter. *Limnol. Oceanogr.* **63**, 1488–1501 (2018). 30166689 10.1002/lno.10786 PMC6108407

[46] Sø, J. S. et al. Ebullition dominates high methane emissions globally across all lake sizes. *Biogeochemistry* **168**, 43 (2025).

[47] Jane, S. F. et al. Widespread deoxygenation of temperate lakes. *Nature* **594**, 66–70 (2021). 34079137 10.1038/s41586-021-03550-y

[48] Jansen, J. et al. Climate-driven deoxygenation of northern lakes. *Nat. Clim. Change* **14**, 832–838 (2024).

[49] Davidson, T. A. et al. Synergy between nutrients and warming enhances methane ebullition from experimental lakes. *Nat. Clim. Change* **8**, 156–160 (2018).

[50] Lewis, A. S. L. et al. Anoxia begets anoxia: a positive feedback to the deoxygenation of temperate lakes. *Glob. Change Biol.* **30**, e17046 (2024). 10.1111/gcb.17046 38273535

[51] Pawlowicz, R. M_Map: a mapping package for MATLAB, version 1.4m. https://www.eoas.ubc.ca/~rich/map.html (2020).

[52] MATLAB, version R2025a (The MathWorks Inc., 2025).

[53] Tan, Z. & Zhuang, Q. Arctic lakes are continuous methane sources to the atmosphere under warming conditions. *Environ. Res. Lett.* **10**, 054016 (2015).

[54] Tan, Z. et al. Modeling CO_2_ emissions from Arctic lakes: model development and site-level study. *J. Adv. Model. Earth Syst.* **9**, 2190–2213 (2017).

[55] Tan, Z. et al. A lake biogeochemistry model for global methane emissions: model development, site-level validation, and global applicability. *J. Adv. Model. Earth Syst.* **16**, e2024MS004275 (2024).

[56] Messager, M. L. et al. Estimating the volume and age of water stored in global lakes using a geo-statistical approach. *Nat. Commun.* **7**, 13603 (2016). 27976671 10.1038/ncomms13603 PMC5171767

[57] Khazaei, B. et al. GLOBathy, the global lakes bathymetry dataset. *Sci. Data* **9**, 36 (2022). 35115560 10.1038/s41597-022-01132-9 PMC8814159

[58] Woolway, R. I. et al. Phenological shifts in lake stratification under climate change. *Nat. Commun.* **12**, 2318 (2021b). 33875656 10.1038/s41467-021-22657-4 PMC8055693

[59] Golub, M. et al. A framework for ensemble modelling of climate change impacts on lakes worldwide: the ISIMIP lake sector. *Geosci. Model Dev.* **15**, 4597–4623 (2022).

[60] Tan, Z., Zhuang, Q. & Walter Anthony, K. Modeling methane emissions from arctic lakes: Model development and site-level study. *J. Adv. Model. Earth Syst.* **7**, 459–483 (2015).

[61] Guo, M. et al. Validation and sensitivity analysis of a 1-D lake model across global lakes. *J. Geophys. Res. Atmos.* **126**, e2020JD033417 (2021).

[62] Guseva, S. et al. Multimodel simulation of vertical gas transfer in a temperate lake. *Hydrol. Earth Syst. Sci.* **24**, 697–715 (2020).

[63] Pilla, R. M. et al. Deeper waters are changing less consistently than surface waters in a global analysis of 102 lakes. *Sci Rep.* **10**, 20514 (2020). 33239702 10.1038/s41598-020-76873-x PMC7688658

[64] Stetler, J. T., Jane, S. F., Mincer, J. L., Sanders, M. N. & Rose, K. C. Long-term lake dissolved oxygen and temperature data, 1941–2018 ver 4. *Environmental Data Initiative*10.6073/pasta/7686da566d3589b95b01c530c8cd0a22 (2024).

[65] Yuan, H., Carpenter, S., Kimura, N., Lathrop, R. & Wu, C. North temperate lakes LTER: spatially distributed water temperature (2004, 2006) and sediment temperature (2006) of Lake Wingra ver 6. *Environmental Data Initiative*10.6073/pasta/83befca04cf9fac2d1111eaf0dc3702f (2022).

[66] Gu, R. & Stefan, H. G. Validation of cold climate lake temperature simulation. *Cold Reg. Sci. Technol.* **22**, 99–104 (1993).

[67] Li, Y., Li, N., Feng, J., Qian, J. & Shan, Y. Temporal temperature distribution in shallow sediments of a large shallow lake and estimated hyporheic flux using VFLUX 2 model. *Water* **13**, 300 (2021).

[68] Schlegel, R. W. & Smit, A. J. heatwaveR: a central algorithm for the detection of heatwaves and cols-spells. *J. Open Source Softw.* **3**, 821 (2018).

[69] Stefan, H. G., Hondzo, M., Fang, X., Eaton, J. G. & McCormick, J. H. Simulated long-term temperature and dissolved oxygen characteristics of lakes in the north-central United States and associated fish habitat limits. *Limnol. Oceanogr.* **41**, 1124–1135 (1996).

[70] Woolway, R. I., Maberly, S. C., Jones, I. D. & Feuchtmayr, H. A novel method for detecting the onset of thermal stratification in lakes from surface water measurements. *Water Resour. Res.* **50**, 5131–5140 (2014).

[71] Read, J. S. et al. Simulating 2368 temperate lakes reveals weak coherence in stratification phenology. *Ecol. Modell.* **291**, 142–150 (2014).

[72] R Core Team. *R: A Language and Environment for Statistical Computing* (R Foundation for Statistical Computing, 2019); https://www.R-project.org/

[73] Woolway, R. I., Shi, H., Tan, Z. & Jansen, J. Dataset and code for ‘Lake sediment heatwaves under global warming’. *Zenodo*10.5281/zenodo.18507326 (2026). 10.1038/s41561-026-01986-3 PMC13259926 42291096

