# Supplementary material for: Lake sediment heatwaves under global warming
Source: Nat Geosci. 2026 Jun 1;19(6):639–45. doi: 10.1038/s41561-026-01986-3 (PMC13259926; doi:10.1038/s41561-026-01986-3)
Supplement: Supplementary file 1 — Supplementary Figs. 1–12. [file 41561_2026_1986_MOESM1_ESM.pdf]

nature geoscience

Supplementary information https://doi.org/10.1038/s41561-026-01986-3

# Lake sediment heatwaves under global warming

In the format provided by the authors and unedited

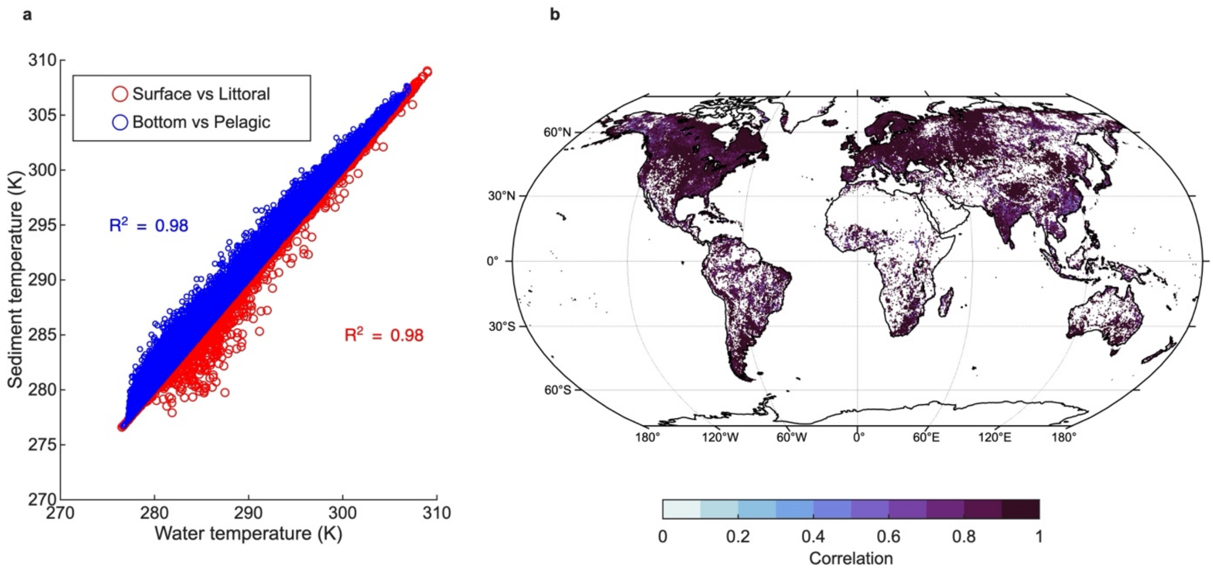


**Supplementary Figure 1.** Shown in (**a**) is a comparison of (i) annual lake surface water temperature and littoral sediment temperature (red), and (ii) annual lake bottom water temperature and pelagic sediment temperature (blue). (**b**) Correlation between lake bottom water temperature and pelagic sediment temperature in the studied lakes. All correlation coefficients were statistically significant ($p < 0.01$).

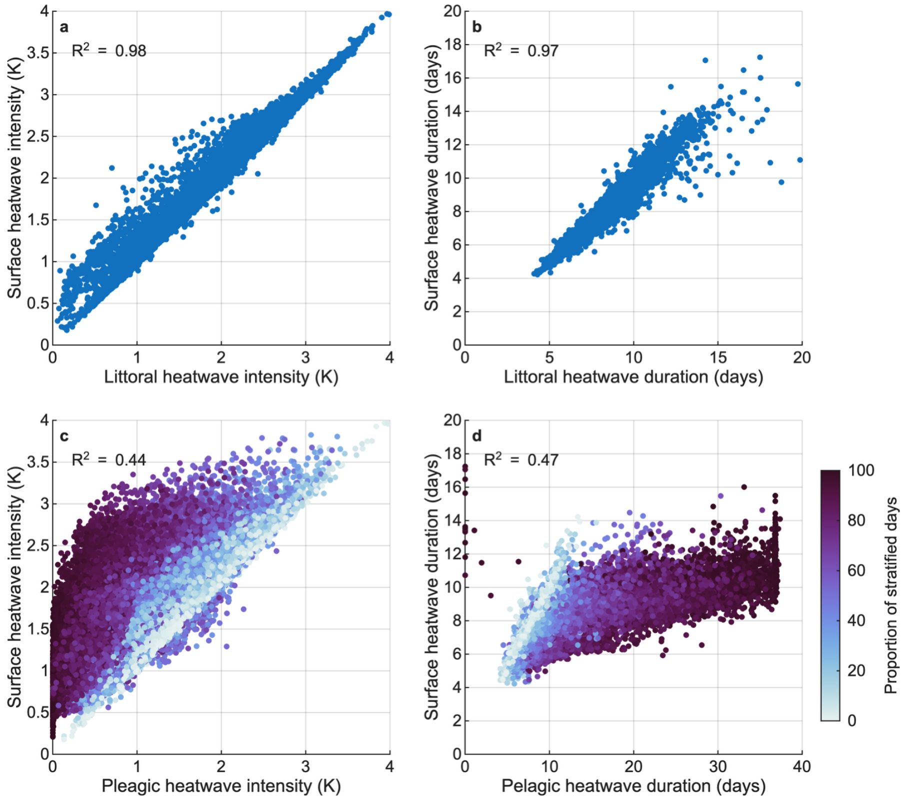


**Supplementary Figure 2.** Shown are the relationships between **a**) the intensity, and **b**) the duration of lake surface heatwaves with littoral sediment heatwaves, and between **c**) the intensity and **d**) duration of surface heatwaves and pelagic sediment heatwaves, as well as the impact of the proportion of annual stratified days.

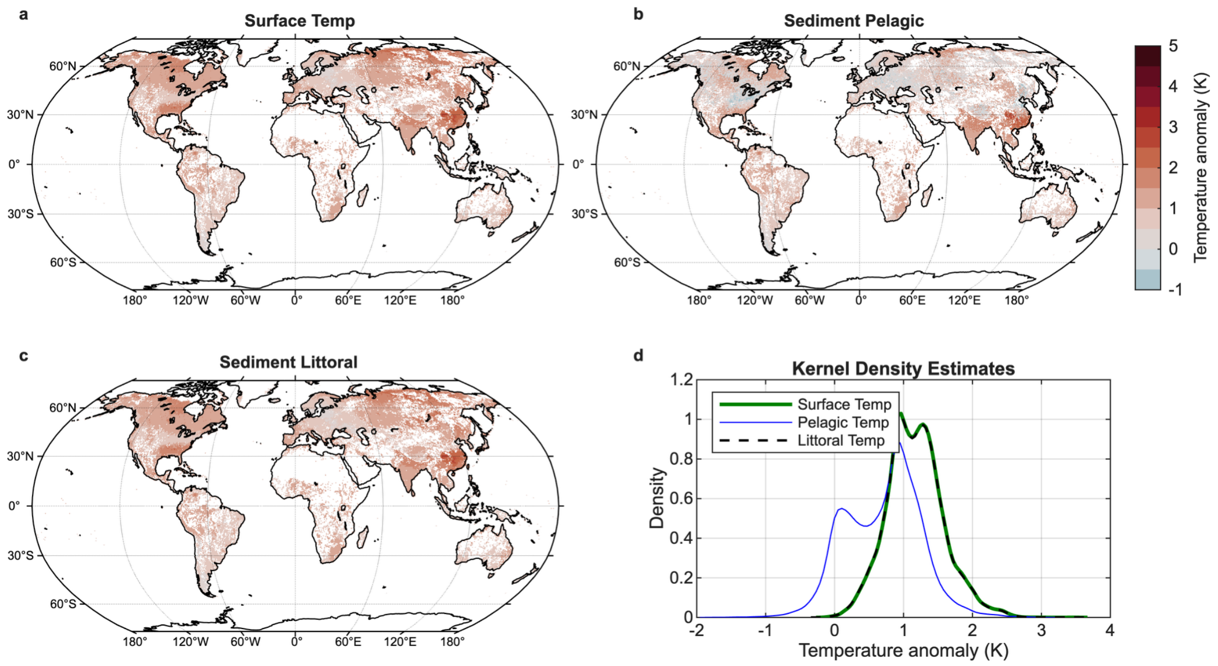


**Supplementary Figure 3.** Future changes in lake surface water temperature, pelagic lake sediment temperature, and littoral lake sediment temperature under SSP126.

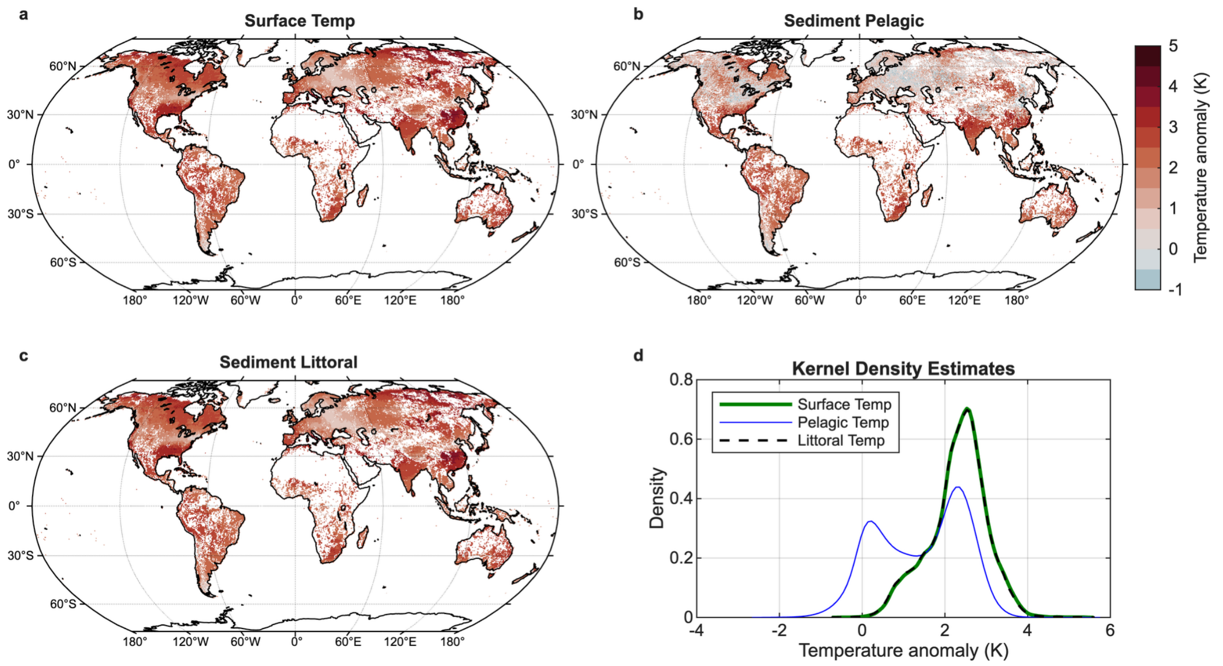


**Supplementary Figure 4.** Future changes in lake surface water temperature, pelagic lake sediment temperature, and littoral lake sediment temperature under SSP370.

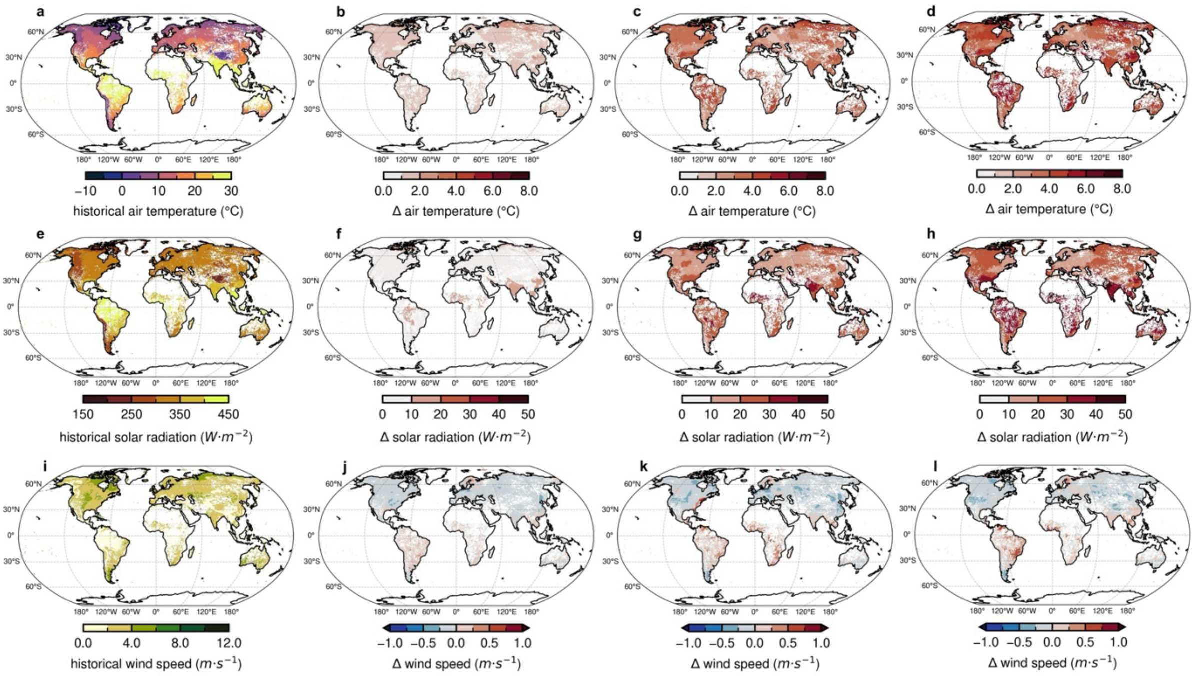


**Supplementary Figure 5.** Historical and future patterns in atmospheric forcing. Shown are global variations in historic and future changes in (**a-d**) air temperature, (**e-h**) solar radiation, and (**i-l**) wind speed. Historical patterns are shown in panels **a, e** and **i**. Future changes are shown for SSP126 (**b, f, j**), SSP370 (**c, g, k**) and SSP585 (**d, h, l**). Future changes refer to differences by the end of the 21st century relative to historic conditions.

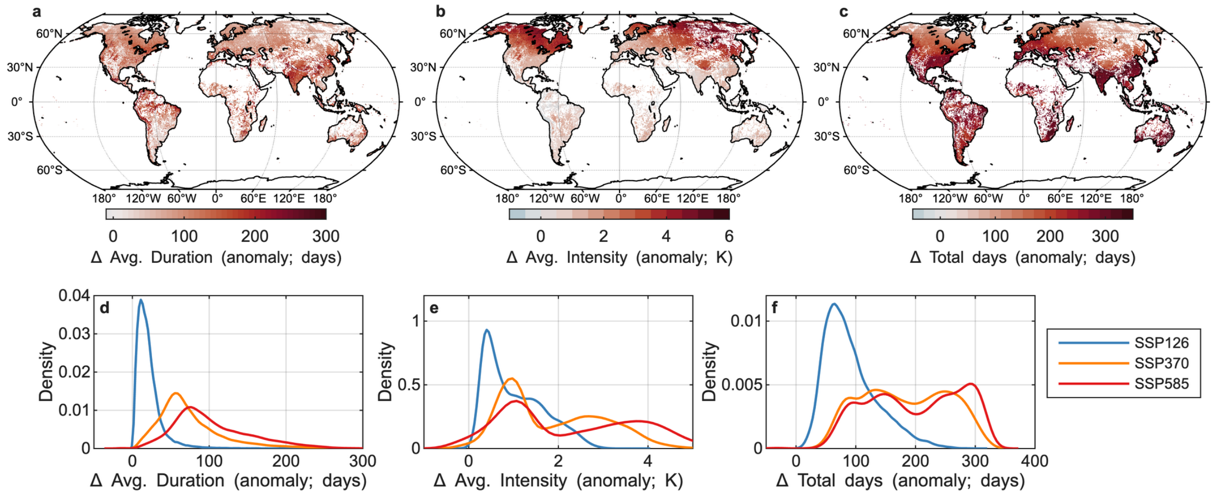


**Supplementary Figure 6.** Future changes in littoral lake sediment heatwaves under future climate change. Shown are the spatial patterns of change under SSP 585 (a-c), and the histogram of simulated changes under SSP126, SSP370, and SSP585.

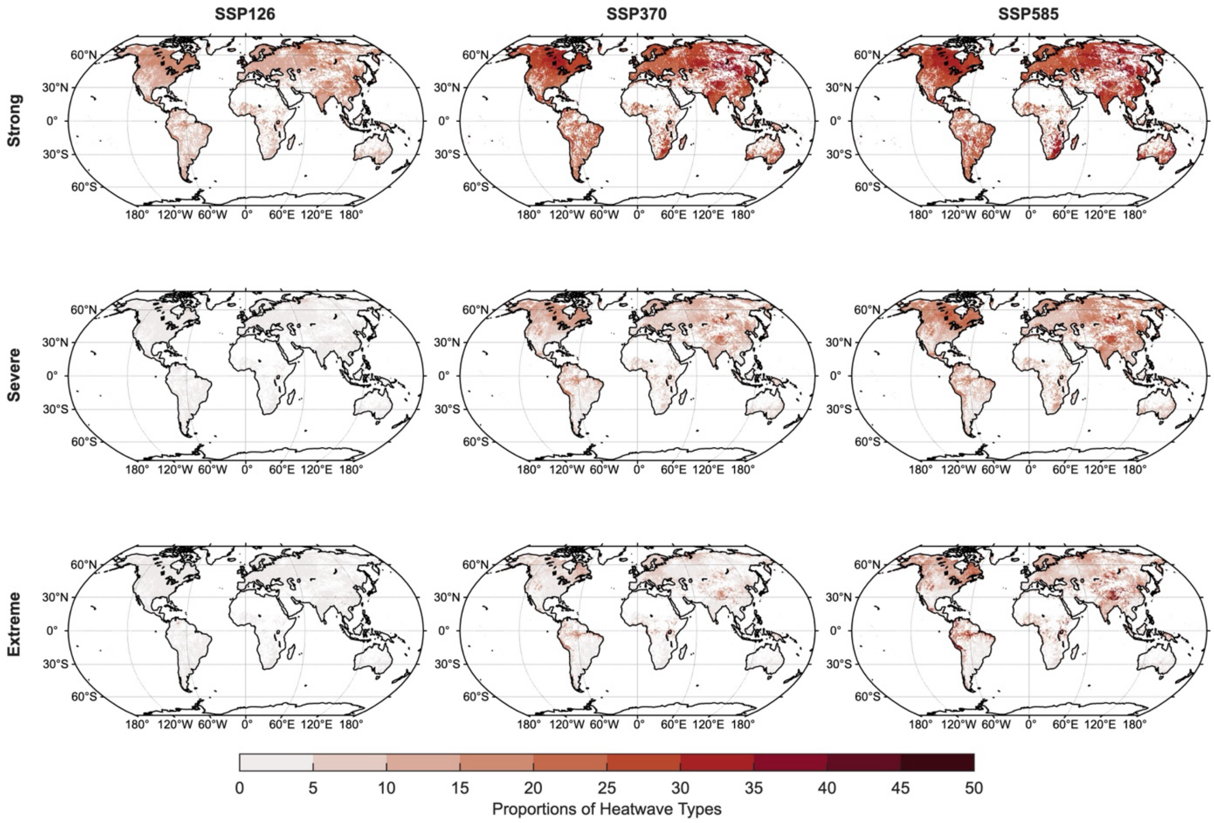


**Supplementary Figure 7.** Shown are the proportion of littoral lake sediment heatwaves globally that are categorised as strong, severe, and extreme in the future (2071-2100). Results are shown under SSP126, SSP370, and SSP585.

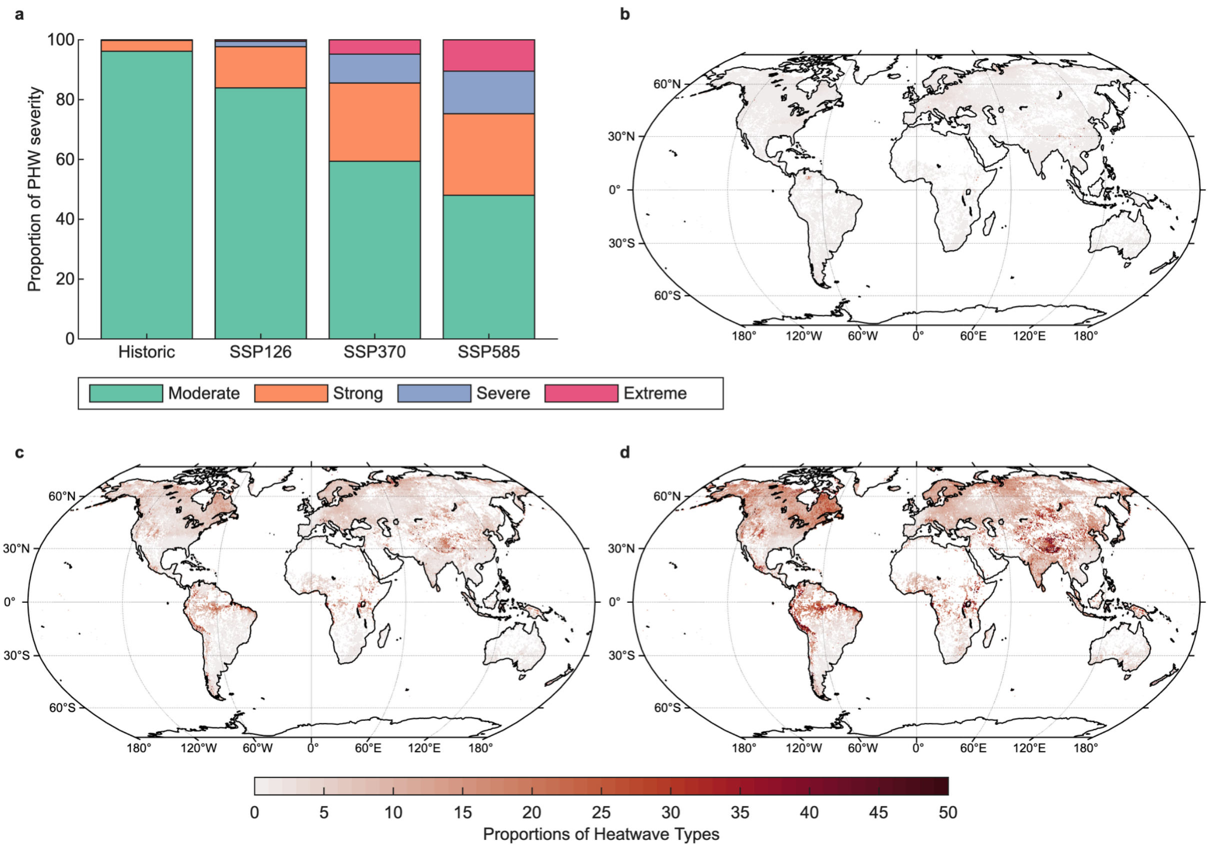


**Supplementary Figure 8.** Shown are (**a**) the proportion of lake sediment (littoral) heatwaves globally that are categorised as moderate, strong, severe, and extreme during the historic (1980-2010) and future (2071-2100) periods. Results for the latter are shown under SSP126, SSP370, and SSP585. In panels **b**, **c**, and **d**, we show the spatial patterns in the proportion of **b**) strong, **c**) severe, and **d**) extreme lake sediment heatwaves under SSP585.

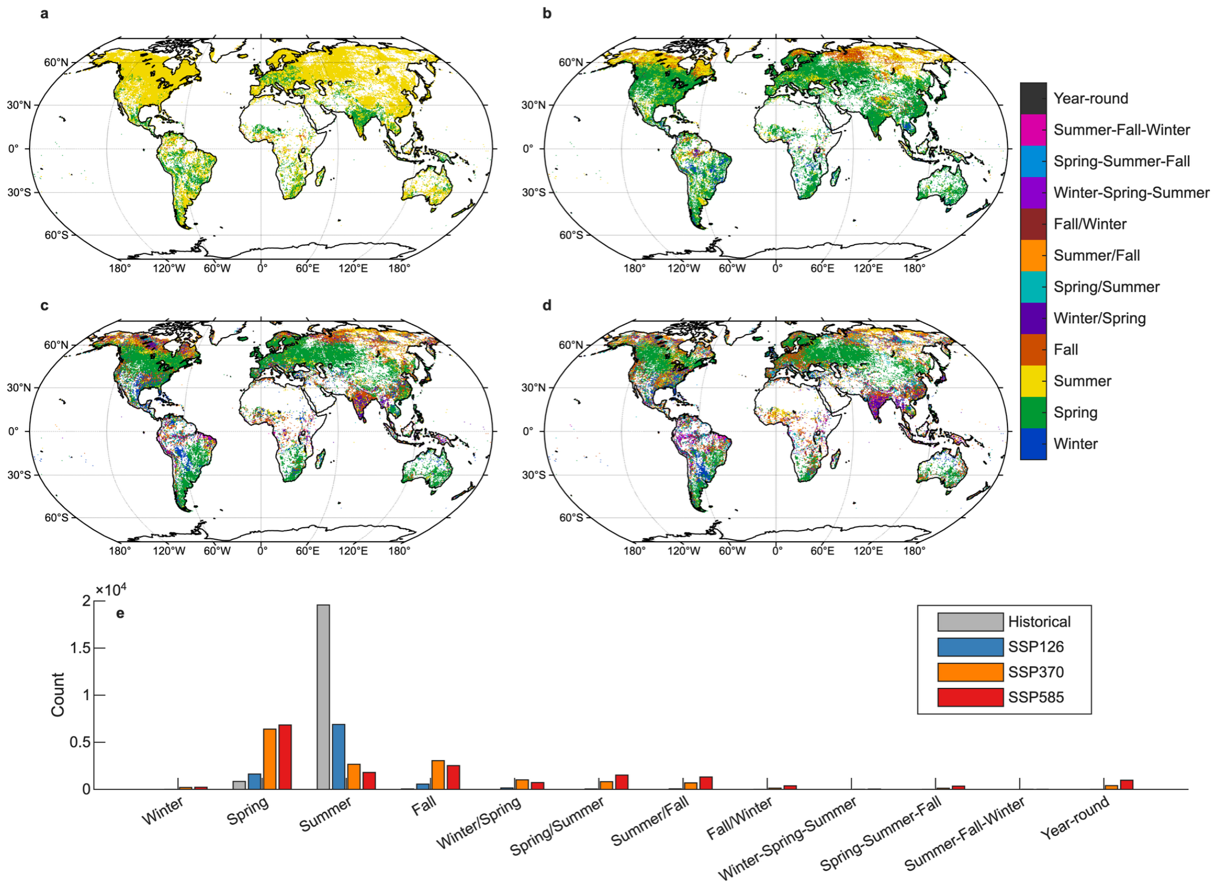


**Supplementary Figure 9.** The seasonality of littoral sediment heatwaves during (**a**) the historical period, and under future climate scenarios (**b**) SSP126, (**c**) SSP370, and (**d**) SSP585. Shown are the season(s) in which pelagic lake sediment heatwaves most commonly occur. Panel (**e**) shows the dominant season (or multiple seasons) in which pelagic lake sediment heatwaves occur in lakes during the historic and future periods.

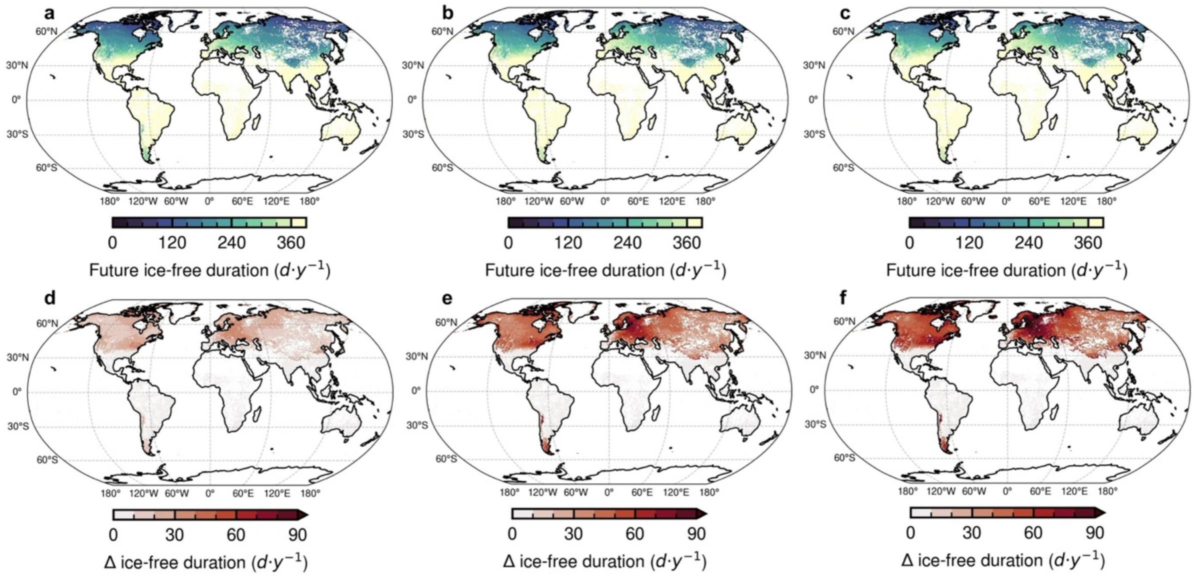


**Supplementary Figure 10.** Future changes in lake ice duration. Shown are the ice durations by the end of the 21st century under (**a**) SSP126, (**b**) SSP370, and (**c**) SSP585. We also compare these future conditions to historic values, represented as the ice duration anomaly (**d-f**).

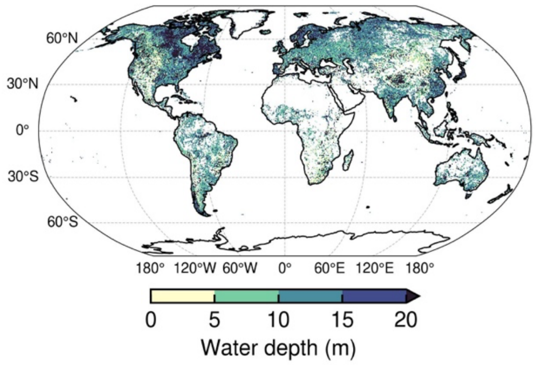


**Supplementary Figure 11.** The representative depth of each grid used in the ISIMIP3b simulations.

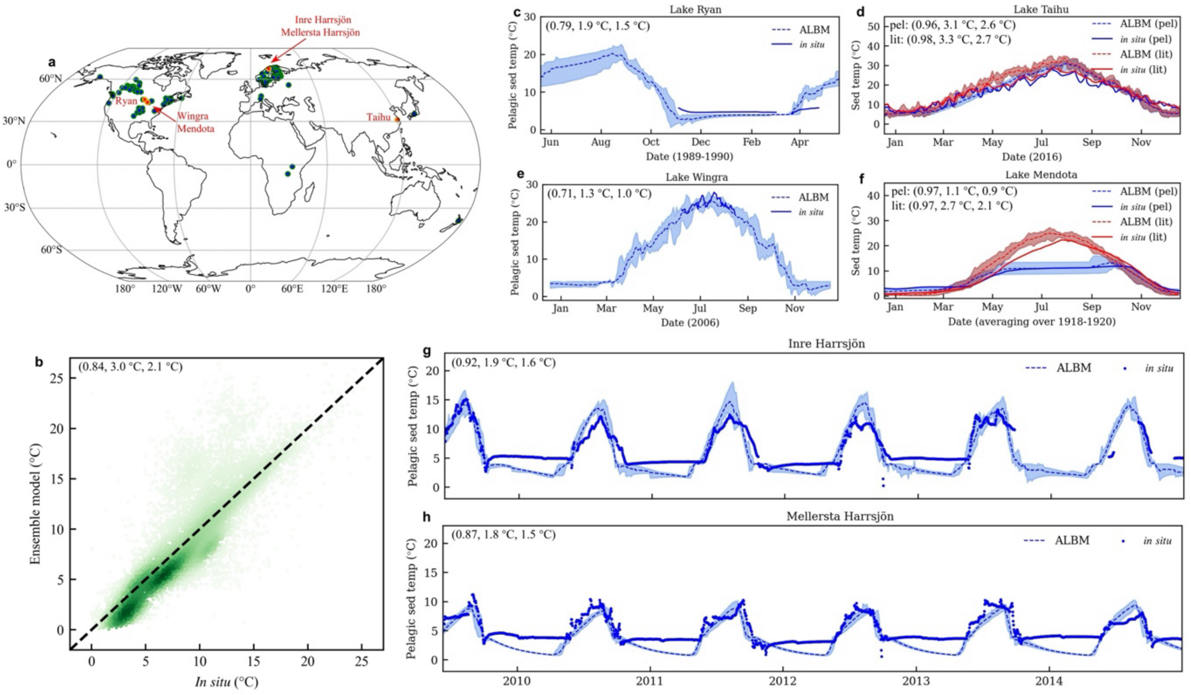


**Supplementary Figure 12.** Validation of ALBM's simulation on water and sediment temperatures. Shown are the distribution of the 138 lakes (green circles) with measurements of lake bottom water temperatures and 6 lakes (red circles) with surface sediment temperatures (**a**); comparison between *in situ* measured and modelled lake bottom water temperature (**b**) and comparison between measured and modelled sediment temperature (**c-h**). Simulation results in (**b**) and the dashed lines in (**c-h**) refer to the mean values averaged over five different GCMs. The darkness of the green color in (**b**) signifies the density of observations. Shaded regions in (**c-h**) indicate the ranges of simulated sediment temperatures under five different GCMs. Evaluation metrics (*R*, *RMSE*, *MAE*) are shown in (**b-h**).
