# Supplementary material for: Lake sediment heatwaves under global warming
Source: Nat Geosci. 2026 Jun 1;19(6):639–45. doi: 10.1038/s41561-026-01986-3 (PMC13259926; doi:10.1038/s41561-026-01986-3)
Supplement: Supplementary file 2 — Peer Review File [file 41561_2026_1986_MOESM2_ESM.pdf]

nature portfolio

Peer Review File

Lake sediment heatwaves under global warming

Corresponding Author: Dr R. Iestyn Woolway

This file contains all reviewer reports in order by version, followed by all author rebuttals in order by version.

Version 0:

Reviewer comments:

Reviewer #1

(Remarks to the Author)
Compared to the extensively studied atmospheric heatwaves and marine heatwaves, lake heatwave is a new research topic and was first proposed by the Pro. Woolway in his 2021 Nature paper. This paper shows the first assessment of lake sediment heatwaves at the global scale, which can be considered as an extension of his 2021 Nature work and definitely deepen our knowledge of heat extremes in inland water bodies. I suggest the paper can be accepted after addressing the following comments.

Major comments:
1. Lines 70-74: I think this is the knowledge gap stated in line 76. However, this is not enough in my opinion. I think knowledge gap is not only the topic which has not been studied yet, but is also related to the technical and scientific barriers why the community have not investigated this (such as lack of in-situ / satellite observations or the incapacity of numerical lake models).

2. Lines 95-99: As we know, there are two lake models (GOTM and ALBM) participating in ISIMIP3b for global lake simulations. Why did you choose ALBM other than GOTM? I think this is related to point 1.

3. Lines 315-330: I suggest the definitions of littoral and pelagic sediment can be described in detail with a schematic figure. Is it possible to extract littoral and pelagic sediment temperatures with other lake model (e.g., GOTM) using hypsographic dataset? If not, this may be another innovative method for studying lake sediment heatwaves.

4. Discussion: This section mainly focuses on the effects of lake sediment temperature or heatwaves on biogeochemical cycles. Is it possible to give some quantitative estimates or assessments?

5. Lines 398-399: This study focuses on ice-free season. Please provide a global distribution of lake ice-free season for the historical and future period. I think this is useful for readers to understand the shift in the time of sediment heatwaves. By the way, in future scenario, the ice-free season would lengthen, especially under SSP585. How about this effect on metrics of lake sediment heatwaves?

Monir comments:
1. Lines 154-157 are the same to lines 160-162.
2. Figure 1: Please add statistical significance test for the two means (littoral and pelagic) in panels g to i. Are the averages of duration, intensity and total days different significantly between two kinds of sediment heatwaves?
3. Figure 4: It is not easy for me to distinguish some colors in panels a to d, such as Fall/Winter vs. Fall, Summer/Fall vs. Summer.
4. Figure S2: Is it possible to add some statistics in scatter plots? If the correlation coefficients in right panel have passed the significance test, please describe this in figure caption.
5. Figure S4: Please add statistics for these scatter plots.
6. Figure S15: Please add statistics (R, RMSE, MAE) for panel b and c.

Reviewer #2

(Remarks to the Author)

The paper used the Advanced Lake Biogeochemistry Model (ALBM) to simulate global lake water and sediment temperature at a spatial resolution of 0.5 x 0.5 degrees. The lake temperature model was driven by several climate models for historic (1901-2014) and future periods (2015-2100) under three SSP climate projection pathways. Historically, littoral sediment heatwaves (pelagic zone heatwaves were also examined) with an intensity of 1.9 K lasted 8.2 days, with the intensity, duration, and frequency expected to increase with climate change. The authors claim that such sediment heatwaves have significant impacts across a wide range of freshwater ecosystems processes such as nutrient loadings, harmful algae blooms, greenhouse gas emissions (methane), aquatic habitat shifts, etc. While this is an interesting topic, I feel that this paper is not suitable for Nature Geoscience for several reasons that I provide below.

The occurrence of a heatwave was determined during the ice-free season using modelled temperature time series climatologies. Using an existing R package, a heatwave occurred when the daily temperature exceeded the 90th percentile. I found nothing wrong with this approach, however, the intensity of the heatwaves (historic and projected) are small, on the order of between 1 to 2 K (average), and the standard deviation was large (+/- 0.6 K). I don't find this result surprising, but I do feel that the motivations for the study as provided in the Introduction, and the points raised in the Discussion and Conclusions are far-reaching given such a small temperature change. In several of the references provided, I could not find any mention of how sediment temperature itself affects a process/organism. In others, the temperature increases required to have a significant impact on a stated processes were greater than the increase in sediment temperature associated with a heatwave reported here. For example, methane production (as many biochemical reactions) increases as Q10 functon(a 10 C rise for an increase in production), and depends not only on temperature but the liable carbon pool (how was that accounted for?). The release of phosphate, nitrogen and other nutrients in the sediment is also often a Q10 function. Many studies that incubate lake sediment find an increase once sediment temperatures increase by 10 or 20 C. These temperature increases that invoke a significant response are several times the magnitude of the sediment heatwaves reported here, and there are many other variables at play that are apparently not accounted for. Although there is little question that temperature does affect these processes provided in a few of the examples, in many instances, the cited literature does not support these changes over the small range in sediment temperature increases during a heatwave. The justification of the study and the discussion points/conclusions should be framed in a realistic scope that is commensurate with the results, not to mention that there are a multitude of factors in addition to temperature that directly influence many of these processes.

My second area of concern is related to the methodology. The paper relies on the use of the lake model (the ALBM), itself driven by other models (GCMs), with those driven by SSP models for climate change projections. Results are at times presented as one prediction against another using the same model, so results are expectedly good. Fig. S2 is an example of this; the comparison of lake surface or bottom temperature to littoral or pelagic sediment temperature is great (as expected) and was therefore used to the justify the use of bottom water temperature as a proxy for sediment temperature (line 349), but this is just a validation of the internal workings of the ALBM model. I fully appreciate the fact that the there are few direct measurements of sediment temperatures available and that the authors did offer a comparison using some measurement from 4 lakes (Fig. S15), however, those data are indeed very limited in geographic location and temporal coverage and therefore scaling from a handful of measurements to the entire globe over time scales of decades is a huge leap.

My final major concern relates to the spatial and temporal variation of sediment thermal and biological properties. Temperature alone cannot explain the likelihood of the occurrence of many of the processes described in the Introduction, Discussion, and Conclusions, and the temperature response can vary regionally and within individual lakes. Methane release requires carbon-rich sediment; thermal stresses vary with species and location. Sediment temperature depends not only on adjacent water temperature (and all the variables that control that), but the sediment's thermal conductivity/heat capacity. Horizontal advection of heat along shorelines with shallow waters could also be important and not represented by a 1-D model. I saw no mention of which values(s) were used, how there were determined, or the sensitivity of sediment temperature to a realistic range in sediment thermal properties. Most lakes, even large, deep ones, have many shallow areas in bays and river inlets, and I wondered how these are resolved in within a 0.5 x 0.5 degree grid.

For these reasons, I feel that this paper is not appropriate for Nature Geoscience and is much better suited for a model-focused journal such as JAMES or Geosci Model Dev. since this is essentially a modelling paper with results and conclusions that are nearly entirely model-based.

A note on the References:

I found that several of the cited papers I could not find the relevant information as referenced in the text. For example:

Line 335: 'For example, ALBM has previously been used to simulate lake temperature, as well as changes to stratification and mixing cycles in lakes, and has been shown to accurately reproduce temporal variations in bottom water AND sediment temperatures (Tan et al., 2015; Woolway et al., 2021b; Golub et al., 2022).'

Tan et al. 2015. This paper deals with Arctic lakes/methane, and list another 2015 paper that does deal with temperature validation as 'at press' (the proper citation is missing). The JAMES paper by Tan, Zhuang, and Anthony (2015) (also on the topic of methane release from pan-Arctic lakes). In this paper I see 1 lake (Goldstream Lake, Alaska) with a comparison between measured and modelled sediment reported for one winter period (Sept/2008 – May/2009) when temperatures were largely in the 4-5 C range.

Woolway et al 2021b - I could not find mention of sediment temperatures.

Golub et al 2022 - I could not find mention of validation/calibration of sediment temperatures.

Reviewer #3

(Remarks to the Author)
The authors did an interesting study investigating lake sediment heatwaves under global warming, which is a large scale study of great impact. Reviewing the whole manuscript, I found several points that might be able to improve: (1) Comparing heatwaves between sediment and lake surface: How about comparing with subsurface lake heatwaves? The authors' study "Subsurface heatwaves in lakes", published at NCC, investigated subsurface lake heatwaves. In my understanding, lake bottom water interacts directly with sediment, thus, it is reasonable to compare heatwaves in these layers. (2) Why these five GCMs are selected? (3) Why omit SSP245 for scenario setup? (4) For model performance, it is better to report the RMSE value as well, a widely used metric to evaluate model performance. (5) Some figures shall be improved in quality and presentation. The detailed comments can be found in the attached file.

Reviewer #4

(Remarks to the Author)
The authors present the first global-scale projection and analysis of lake sediment heatwaves, including both historical and future periods, using an extensive dataset. The Introduction and Results sections are well written, and the figures are generally clear and appealing. My comments mainly concern the Methods/Discussion sections, along with several minor points detailed below.

Methodological Comments
Line 300
Is the bathymetry lake-specific, or is a representative bathymetry used for all lakes? Clarification is needed here. Bathymetry can influence the spatial discretization of the model grid, the definition of littoral versus pelagic zones, and lake stratification processes. If a representative bathymetry is applied to all lakes, this choice should be made explicit and ideally illustrated in the Supporting Information for transparency.

Lines 318–323
The model grid configuration is not sufficiently clear. A schematic diagram illustrating the lake and sediment discretization would be helpful. I consulted the original ALBM model paper, where I understand that N sediment columns can be used — could the authors explain the choice of using four columns specifically?
Additionally, the definition of littoral and pelagic zones requires clarification. Is the same spatial definition applied for all lakes regardless of depth? Are littoral sediments assumed to be alternately exposed to the atmosphere and submerged (i.e., forced alternately by atmospheric conditions and water temperature), or are they always submerged? Related to this, was seasonal lake-surface-area variability (wet/dry periods) considered?
Regarding vertical discretization, stating the use of “40 sediment layers and 50 water layers” does not adequately convey the true spatial resolution unless layer thickness is also specified. Given that results and validation are presented at 0.5-m or 1-m sediment depths, is this resolution achievable with the applied discretization, particularly for deep lakes where layer thickness may exceed these values?
This also raises the question of why 1 m depth is chosen for the main analyses. Is sediment at 1 m depth still considered representative of sediment surface temperature?

Line 363
Validation against bottom-water temperature may demonstrate model skill for predicting pelagic surface sediment temperature, but it does not necessarily test performance for littoral sediments — particularly if these sediments experience alternating exposure and submergence. If littoral sediments are assumed to remain permanently submerged, this should be clarified explicitly in the Methods section.

Lines 393–394
It appears that future heatwaves may be defined relative to the present baseline (1991–2020). If this interpretation is correct, it would explain why heatwave conditions persist year-round in some future scenarios. However, clarification is needed here. If present-day heatwaves are defined relative to a present baseline, it seems equally reasonable that future heatwaves could be defined relative to a future baseline as well. The authors should clarify which baseline is used for future heatwave identification and explain the rationale for this choice, as this definition underpins all subsequent analyses.

Line 441
Further details on the cross-correlation function would help readers understand precisely how time lags are calculated.

Minor Textual Issues
Lines 152–157 and 160–164
These sections appear to be repetitive.

Forcing Data & Interpretation
Line 172
The projected future results presumably depend strongly — or even entirely — on the meteorological forcing data, yet no information on these forcings is provided. At minimum, summary descriptions of projected air temperature, solar radiation, or

other relevant drivers should be included to help readers understand the physical reasons behind the projected changes in sediment heatwaves and to improve transparency.

Line 221
Following the previous point, the Discussion focuses primarily on the ecological and biogeochemical implications of future sediment heatwaves. Additional discussion of the modeling results themselves would be valuable — including discussion of the projected forcing trends, model uncertainty and sensitivity, and justification of key methodological choices.

Comments on Figures
• Figure 1h — The small panel and the global map are not well aligned with each other (e.g., relative to panels g and i).
• Figure S2a — Surface water temperature appears consistently higher than littoral sediment temperature, while bottom-water temperature appears lower than pelagic sediment temperature. This is interesting and merits further explanation if not already discussed.
• Figure S3 — Only panel (d) includes “Scatter” in the title, whereas panels (b) and (c) do not. For consistency, it would be preferable to standardize the panel titles.
• Figure S15c — In the Lake Mendota panel, the legend entry “insitu (lit)” is missing the left parenthesis.
Additionally, model uncertainty, sensitivity, and any systematic deviations observed during validation should be discussed in the Discussion section.

Version 1:

Reviewer comments:

Reviewer #1

(Remarks to the Author)

Reviewer #2

(Remarks to the Author)
I thank the authors for their careful revisions and respectful responses to my concerns. I find the paper is much improved, and is now suitable for publication.

Reviewer #3

(Remarks to the Author)
The authors have addressed all my concerns. I don't have any further comments. If the other reviewers are satisfied with the revision, the paper is acceptable in my opinion.

Reviewer #4

(Remarks to the Author)
The authors answered my questions quite clearly and added the required explanations in the updated manuscript, especially regarding the reasons for their methodological choices and the discussion of limitations and uncertainties in their results. The newly added section provides a clear description of the Arrhenius-based framework used to quantify heatwave impacts on sediment biogeochemical rates. However, several points require clarification or refinement to ensure mathematical correctness, internal consistency, and appropriate interpretation.

---

1. Clarification of the Arrhenius formulation (Eq. 1)
Equation (1) represents a normalized Arrhenius-type temperature response using an empirical activation energy E_a' expressed in eV, together with Boltzmann’s constant k_B. This formulation is appropriate and internally consistent. However, I think E_a' should be described as the slope of the relationship between $\ln J$ (or equivalently $\ln(J/J(T_c))$) and $1/T$, rather than the slope of the logarithm of a centered rate difference $(J(T_c)-J(T))$. The Arrhenius relationship is linear in the logarithm of the rate (or rate ratio), not in the logarithm of a rate difference. Please correct me if my understanding is wrong.
In addition, the authors state that T_c is 16.8 °C and 13.4 °C in the littoral and pelagic zones, respectively. It should be noted whether these two values are derived from model output (if that is the case). It should also be explicitly stated that both T and T_c are converted to Kelvin prior to evaluation. While this may be implicit, stating it clearly would avoid ambiguity.

---

2. Definition of heatwave temperature forcing and its implications
The manuscript defines heatwave intensity as the mean temperature anomaly relative to the seasonal climatology during heatwave events, calculated exclusively over the ice-free season. In Eq. (1), the temperature used to compute the Arrhenius response is defined as:
$$T=T_{\text{historic ice-free mean}}+\overline{(I_{\text{HW}})}$$

This represents a single “effective heatwave temperature” constructed from a long-term mean plus an event-based anomaly. While this is a pragmatic simplification, it should be emphasized that this approach estimates the Arrhenius response

evaluated at an adjusted mean temperature, rather than the true mean reaction rate under temporally varying temperatures during heatwaves.
Because Arrhenius functions are nonlinear, the rate evaluated at a mean temperature generally differs from the mean of rates evaluated over fluctuating temperatures. As such, this approach provides an effective or first-order estimate of heatwave impacts, but may underestimate the contribution of peak temperatures during heatwaves. Explicitly acknowledging this approximation would strengthen the methodological transparency. Discussion is, of course, welcome if my understanding is not correct.

---

3. Consistency between heatwave intensity and duration weighting (Eq. 2)
Equation (2) computes the annual mean rate multiplier as a duration-weighted average, assuming:
a normalized rate of 1 during non-heatwave days, and
a rate of J(T) during heatwave days.
Given that heatwave intensity is defined as a mean anomaly conditional on heatwave events (rather than an annual mean anomaly), this formulation is mathematically consistent, provided that:
$\overline{I_{\mathrm{HW}}}$ is calculated only over heatwave days, and
$t_{\mathrm{hw,tot}}$ represents the total number of heatwave days in the year.
It would nevertheless be helpful to explicitly state this conditionality to avoid confusion and to clarify that intensity and duration are not double-counted. In addition, the assumption that non-heatwave days have a fixed normalized rate of 1 implies that broader background warming outside heatwaves is not considered; this assumption should be stated explicitly and briefly justified.

---

4. Interpretation of “rates” as relative multipliers
The computed quantities J(T)and J_{tot} represent dimensionless relative rate multipliers rather than absolute methanogenesis or respiration rates. This distinction is explained later in the text, but to avoid misinterpretation, it may be clearer to consistently refer to these quantities as “relative rates,” “rate multipliers,” or “fractional temperature effects,” rather than simply “rates.”

---

5. Choice and uncertainty of activation energy values
The use of globally synthesized $E_a^{'}$values for methanogenesis and organic carbon mineralization is appropriate for a global-scale analysis. However, given the known variability in apparent activation energies across substrates, redox conditions, and microbial pathways, a brief discussion of uncertainty or sensitivity to $E_a^{'}$would strengthen the robustness of the conclusions. Even a short acknowledgment of this limitation would be sufficient.

Version 2:

Reviewer comments:

Reviewer #1

(Remarks to the Author)

Reviewer #4

(Remarks to the Author)
Thank you for your detailed responses to the previous round of review. I have no further major concerns.

I only suggest a minor clarification regarding the statistical description of the extended data figure 3&4:

The statistical tests used appear generally appropriate (Wilcoxon rank-sum tests for comparisons between groups). However, the figure legends do not define the elements of the boxplots. In particular, the meaning of the boxes and whiskers (e.g., median, interquartile range, and whisker definition such as 1.5×IQR or min–max) should be explicitly stated, as required by the journal guidelines that all error bars must be defined in the legend.

In addition, Extended Data Figure 4 presents multiple pairwise comparisons among four categories. The legend indicates that Wilcoxon rank-sum tests were used, but it does not specify whether any correction for multiple comparisons (e.g., Holm or Bonferroni adjustment) was applied. This should be clarified.

It would also be helpful to report the sample size (n) for each group in the figure legend.

Open Access This Peer Review File is licensed under a Creative Commons Attribution 4.0 International License, which permits use, sharing, adaptation, distribution and reproduction in any medium or format, as long as you give appropriate credit to the original author(s) and the source, provide a link to the Creative Commons license, and indicate if changes were made.
In cases where reviewers are anonymous, credit should be given to 'Anonymous Referee' and the source.
The images or other third party material in this Peer Review File are included in the article's Creative Commons license, unless indicated otherwise in a credit line to the material. If material is not included in the article's Creative Commons license and your intended use is not permitted by statutory regulation or exceeds the permitted use, you will need to obtain permission directly from the copyright holder.
To view a copy of this license, visit https://creativecommons.org/licenses/by/4.0/

Reviewer #1:
Compared to the extensively studied atmospheric heatwaves and marine heatwaves, lake heatwave is a new research topic and was first proposed by the Pro. Woolway in his 2021 Nature paper. This paper shows the first assessment of lake sediment heatwaves at the global scale, which can be considered as an extension of his 2021 Nature work and definitely deepen our knowledge of heat extremes in inland water bodies. I suggest the paper can be accepted after addressing the following comments.
We thank the reviewer for their positive and insightful assessment. We appreciate the recognition that lake heatwaves represent a new research frontier, and that this study provides the first global-scale assessment of sediment heatwaves in lakes. We have carefully addressed all comments and suggestions raised by the reviewer to further strengthen the clarity, robustness, and presentation of the manuscript.

Major comments:
1. Lines 70-74: I think this is the knowledge gap stated in line 76. However, this is not enough in my opinion. I think knowledge gap is not only the topic which has not been studied yet, but is also related to the technical and scientific barriers why the community have not investigated this (such as lack of in-situ / satellite observations or the incapacity of numerical lake models).
We thank the reviewer for this constructive comment. We agree that a knowledge gap should be articulated not only in terms of what has not yet been studied, but also why it has remained unexplored. In response, we have revised the Introduction to more clearly explain the technical and scientific barriers that have historically prevented the investigation of lake sediment heatwaves.

Specifically, we now clarify that, unlike atmospheric and surface-water heatwaves, sediment heatwaves cannot be directly observed at large spatial scales due to fundamental observational limitations. Satellite remote sensing is restricted to the water surface and provides no information on subsurface or sediment thermal extremes, while long-term, spatially extensive in situ sediment temperature measurements are extremely scarce and geographically limited. As a result, there has been no observational basis to detect, characterize, or compare sediment heatwaves across regions or climate regimes. In addition, we note that until recently, numerical lake models have primarily been developed and evaluated to represent the thermal dynamics of lake water, stratification, and ice phenology, with limited emphasis on sediment thermal processes at global scales. This has further constrained the ability of the community to assess sediment heat extremes in a consistent and mechanistic framework.

We therefore emphasize that the novelty of this study lies in overcoming these limitations by applying a process-based global lake modelling approach to provide the first systematic assessment of lake sediment heatwaves and their future evolution under climate change.

2. Lines 95-99: As we know, there are two lake models (GOTM and ALBM) participating in ISIMIP3b for global lake simulations. Why did you choose ALBM other than GOTM? I think this is related to point 1.
We thank the reviewer for this comment. Within ISIMIP3b, we selected ALBM because it explicitly simulates vertically resolved sediment temperature profiles, which are essential for identifying and characterizing sediment heatwaves. In contrast, other lake models participating in ISIMIP3b, notably GOTM, are designed to simulate water-column thermal dynamics and turbulence and do not natively represent temperature within sediment layers. While GOTM provides bottom water temperature at the sediment–water interface, it does not simulate internal sediment temperature profiles, which are required for this study. We have clarified this rationale and the associated model choice in the Methods section.

3. Lines 315-330: I suggest the definitions of littoral and pelagic sediment can be described in detail with a schematic figure. Is it possible to extract littoral and pelagic sediment

temperatures with other lake model (e.g., GOTM) using hypsographic dataset? If not, this may be another innovative method for studying lake sediment heatwaves.
We thank the reviewer for this helpful suggestion. We agree that a schematic would improve clarity and will include a figure illustrating the definitions of littoral and pelagic sediments. Such schematic diagram is provided in fig S1 of Tan et al., (2024) – see below. We now specifically refer to this figure in the text.

Some specific details of the configuration of water and sediment columns in ALBM is illustrated in the schematic. The littoral zone is defined by the uppermost sediment column (i.e., "Sediment Column 1") which interacts with the shallowest water layers and the pelagic zone is defined by the lowest sediment column (i.e., "Sediment Column N") which interacts with the deepest water layers.

Regarding the second point, it is not possible to extract littoral and pelagic sediment temperatures using other lake models such as GOTM, even when combined with hypsographic datasets, because, to our knowledge, these models do not simulate sediment thermal dynamics or sediment temperature profiles. This capability is specific to ALBM. We have clarified this in the Methods section.

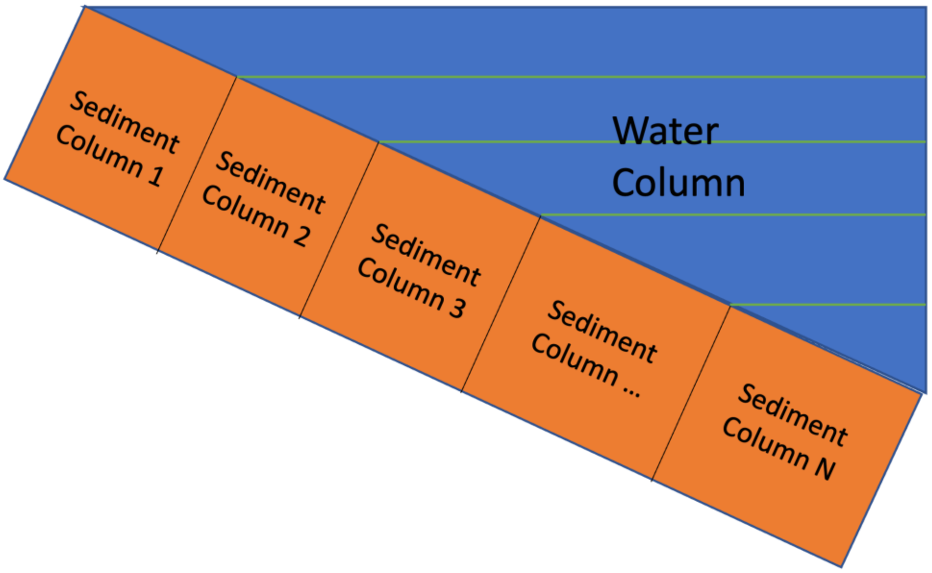

Figure R1. Schematic of the bathymetry-resolved lake structure in ALBM (Fig S1 from Tan et al., 2024).

4. Discussion: This section mainly focuses on the effects of lake sediment temperature or heatwaves on biogeochemical cycles. Is it possible to give some quantitative estimates or assessments?
We thank the reviewer for this valuable suggestion. In response, we have added a quantitative assessment of the potential temperature-driven sensitivity of sediment biogeochemical processes during sediment heatwaves. Specifically, we now estimate the relative change in temperature-dependent reaction rates using an Arrhenius-type exponential temperature response function, based on empirically derived activation energies from global syntheses of lake sediment incubation experiments (Yvon-Durocher et al., 2014; Gudasz et al., 2010).

This approach allows us to quantify the fractional change in biogeochemical process rates attributable solely to sediment temperature anomalies during heatwaves, without attempting to simulate absolute fluxes. Producing more sophisticated and mechanistic projections would require additional information on sediment carbon availability, redox conditions, microbial community composition, and nutrient pools, as well as their spatial variability across lakes - data that are currently not available at the global scale. We therefore deliberately avoid

overemphasizing these quantitative estimates and instead present them as indicative of potential temperature sensitivity rather than as predictive outcomes. These results are now presented in the paper.

In addition, we have revised the Discussion to more clearly distinguish between quantitative temperature-based assessments and broader qualitative implications, and we have softened the wording throughout to ensure that the scope of our conclusions is commensurate with the level of process representation. We believe this balanced approach maintains scientific rigor while highlighting priorities for future research needed to fully resolve biogeochemical responses to sediment heatwaves.

This approach is described in the Methods section as:

We used an Arrhenius-type exponential temperature function (Yvon-Durocher et al., 2014) characterizing the overall temperature sensitivity of methanogenesis and respiration to project rates in lake sediments under historic and future sediment heatwaves:

$$J(T) = J(T_c)e^{E_a'\left(\frac{1}{k_B T_c} - \frac{1}{k_B T}\right)} \qquad (1)$$

where J is the methanogenesis or respiration rate, T is the temperature in K, E_a' is the empirical activation energy in electron volts (eV) and k_B is the Boltzmann constant (8.62×10^{-5} eV K^{-1}). E_a' is the temperature sensitivity of the rate, and is equivalent to the slope of the natural logarithms of the centered rate $(J(T_c) - J(T))$ and temperature $(1/k_B T_c - 1/k_B T)$, where $J(T_c)$ is the rate at a midpoint temperature T_c. We used robust E_a' values for methanogenesis (E_a' = 0.96 eV, Yvon-Durocher et al., 2014) and organic carbon mineralization (E_a' = 0.65 eV, Gudasz et al., 2010) from global syntheses of lake sediment incubation measurements. T is the sum of the historic ice-free mean sediment temperature and the mean heatwave intensity in each 0.5° grid cell. T_c is the global historic ice-free mean sediment temperature, 16.8 °C and 13.4 °C in the littoral and pelagic zone, respectively. Using Eq. 1, we computed rates (J(T)) as departures from an arbitrary historical global mean rate ($J(T_c)$ = 1) (Jansen et al., 2022). By computing dimensionless rates, we obtained relative, direct temperature effects and omitted the need for biological modulating variables required to model absolute rates (e.g. lake trophic state or sediment organic carbon content) (Tan et al., 2024), which are not yet available as a global gridded dataset. In other words, J(T) represents the fractional change in methanogenesis and respiration rates as a function of the departure from T_c. The change in annual mean rates also factors in the duration of heatwaves.

$$J_{tot} = \frac{t_{hw,tot}}{365}(J(T) - 1) + 1 \qquad (2)$$

where J_{tot} is the fractional change in annual mean methanogenesis or respiration rates, assuming a rate of 1 during non-heatwave days, and $t_{hw,tot}$ is the total annual number of heatwave days.

-

In the results section, we describe:

To quantify the impact of lake heatwaves on the rate of biochemical processes in lake sediments, we computed fractional increases in sediment methanogenesis and respiration rates using an Arrhenius-type temperature sensitivity equation (Eq. 1, Methods) (Gudasz et al., 2010, Yvon-Durocher et al., 2014, Jansen et al., 2022). In the shallow littoral zone, from where most CH_4 is typically emitted (Juutinen et al., 2003, Wik et al., 2013), methane production more than doubled during future sediment heatwaves, increasing by a factor of

2.06, 2.22 and 2.28 under SSP 1-2.6, 3-7.0 and 5-8.5, respectively (Table S1). Considering that heatwaves affect a portion of the year (Eq. 2, Methods), we estimated the total increase in methane production at a factor of 1.47, 1.92 and 2.01, respectively (Table S2). Pelagic sediment respiration, an often-dominant driver of seasonal oxygen depletion in lake bottom waters (Livingstone & Imboden, 1996), increased by a factor of 1.47, 1.52 and 1.53 under SSP 1-2.6, 3-7.0 and 5-8.5, respectively (Table S1). Taking into account heatwave duration, total sediment respiration increased by a factor 1.26, 1.41 and 1.41, respectively (Table S2).

-

Table S1. Global mean fractional change in lake sediment methanogenesis and respiration rates during historic and future sediment heatwaves, relative to the rate at the historic ice-free mean sediment temperatures (Eq. 1). 95% confidence intervals are bias-corrected and accelerated (BCa) intervals computed using 5000 replicates.

		Methanogenesis			Respiration		
**Zone**	**Scenario**	**mean**	**95% CI**	**range**	**mean**	**95% CI**	**range**
Littoral	Historic	1.89	1.87-1.91	0.16-11.74	1.40	1.39-1.41	0.29-5.30
	Future, SSP 1-2.6	2.06	2.04-2.08	0.18-12.21	1.50	1.49-1.51	0.31-5.44
	Future, SSP 3-7.0	2.22	2.20-2.24	0.20-12.19	1.59	1.58-1.60	0.33-5.44
	Future, SSP 5-8.5	2.28	2.26-2.30	0.21-12.21	1.63	1.62-1.64	0.34-5.44
Pelagic	Historic	1.92	1.90-1.95	0.26-15.19	1.39	1.37-1.40	0.40-6.31
	Future, SSP 1-2.6	2.09	2.06-2.11	0.27-16.04	1.47	1.46-1.48	0.41-6.55
	Future, SSP 3-7.0	2.16	2.14-2.18	0.27-16.56	1.52	1.51-1.53	0.41-6.69
	Future, SSP 5-8.5	2.18	2.15-2.20	0.27-16.12	1.53	1.52-1.55	0.41-6.57

Table S2. Global mean fractional change in total annual lake sediment methanogenesis and respiration due to sediment heatwaves, i.e. considering the duration of heatwaves (Eq. 2). 95% confidence intervals are bias-corrected and accelerated (Bca) intervals computed using 5000 replicates.

		Methanogenesis			Respiration		
**Zone**	**Scenario**	**mean**	**95% CI**	**range**	**mean**	**95% CI**	**range**
Littoral	Historic	1.08	1.07-1.08	0.93-1.97	1.04	1.04-1.04	0.94-1.39
	Future, SSP 1-2.6	1.47	1.46-1.48	0.40-8.29	1.23	1.22-1.23	0.51-4.05
	Future, SSP 3-7.0	1.92	1.90-1.94	0.29-10.43	1.45	1.44-1.45	0.42-4.86
	Future, SSP 5-8.5	2.01	1.99-2.03	0.26-10.41	1.50	1.49-1.50	0.39-4.81
Pelagic	Historic	1.09	1.09-1.09	0.72-1.63	1.04	1.04-1.04	0.72-1.63
	Future, SSP 1-2.6	1.57	1.56-1.59	0.51-11.91	1.26	1.26-1.27	0.62-5.10
	Future, SSP 3-7.0	1.87	1.85-1.89	0.39-15.05	1.41	1.40-1.41	0.51-6.19
	Future, SSP 5-8.5	1.86	1.85-1.88	0.37-14.69	1.41	1.40-1.41	0.50-6.04

5. Lines 398-399: This study focuses on ice-free season. Please provide a global distribution of lake ice-free season for the historical and future period. I think this is useful for readers to understand the shift in the time of sediment heatwaves. By the way, in future scenario, the ice-free season would lengthen, especially under SSP585. How about this effect on metrics of lake sediment heatwaves?
We thank the reviewer for this insightful suggestion. In response, we have added a global map of ice-free season length for both the historical period (1981–2010) and the future period (2071–2100) under SSP585 (Fig. S18). This allows readers to visually assess spatial patterns and projected changes in lake ice cover, which directly influence the timing and occurrence of sediment heatwaves.

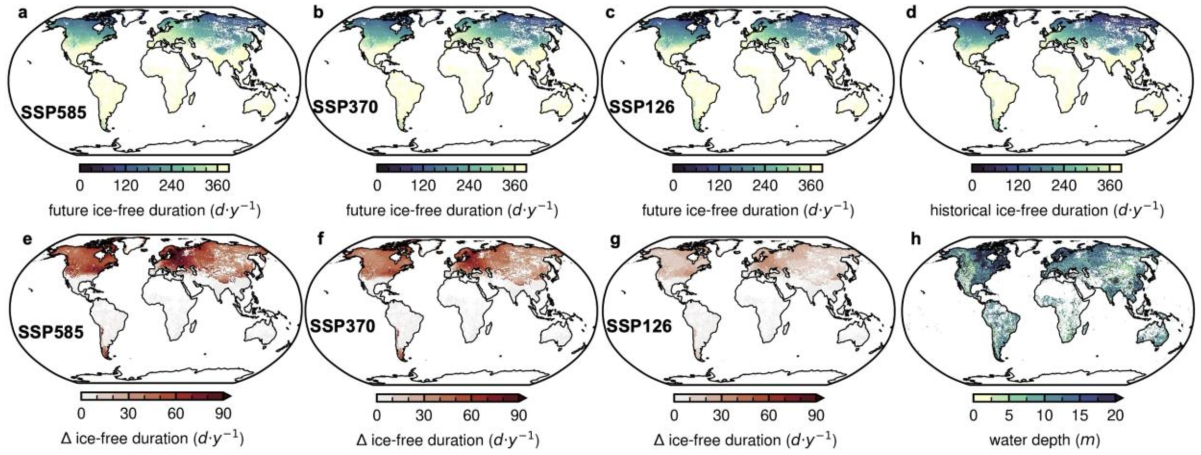

Figure S18 | Future changes in lake ice duration. Shown are the ice durations by the end of the 21st century under (a) SSP126, (b) SSP370, and (c) SSP585. We also compare these future conditions to historic (d) conditions, represented as the ice duration anomaly (e-g). In panel h, we show the representative depth of each grid, used in the ISIMIP3b simulations.

We also added a discussion highlighting that the projected lengthening of the ice-free season, particularly under high-emission scenarios such as SSP585, is expected to extend the period during which sediment heatwaves can develop. Specifically, longer ice-free periods may increase the duration and frequency of sediment heatwaves, as well as cumulative metrics such as total heatwave days and integrated intensity.

Monir comments:
1. Lines 154-157 are the same to lines 160-162.
Apologies for this oversight. This has now been corrected.

2. Figure 1: Please add statistical significance test for the two means (littoral and pelagic) in panels g to i. Are the averages of duration, intensity and total days different significantly between two kinds of sediment heatwaves?
We have modified this figure accordingly. We decided to move the boxplots to a separate figure (Fig. S4) to ensure that the figures were as clear as possible (as requested by reviewer 3). We tested the significance of the differences between littoral and pelagic sediment heatwave metrics using the Wilcoxon rank-sum test.

3. Figure 4: It is not easy for me to distinguish some colors in panels a to d, such as Fall/Winter vs. Fall, Summer/Fall vs. Summer. OK.
We thank the reviewer for noting the difficulty in distinguishing some seasonal categories. We revised the colormap to enhance perceptual contrast between similar seasons (e.g., Fall vs. Fall/Winter and Summer vs. Summer/Fall) and ensured all categories are more clearly distinguishable.

4. Figure S2: Is it possible to add some statistics in scatter plots? If the correlation coefficients in right panel have passed the significance test, please describe this in figure caption.
Yes, these have now been added. We confirm that the correlation coefficients are all statistically significant.

5. Figure S4: Please add statistics for these scatter plots.
Statistics have now been added.

6. Figure S15: Please add statistics (R, RMSE, MAE) for panel b and c.
Statistics have now been added.

Reviewer #2:
The paper used the Advanced Lake Biogeochemistry Model (ALBM) to simulate global lake water and sediment temperature at a spatial resolution of 0.5 x 0.5 degrees. The lake temperature model was driven by several climate models for historic (1901-2014) and future periods (2015-2100) under three SSP climate projection pathways. Historically, littoral sediment heatwaves (pelagic zone heatwaves were also examined) with an intensity of 1.9 K lasted 8.2 days, with the intensity, duration, and frequency expected to increase with climate change. The authors claim that such sediment heatwaves have significant impacts across a wide range of freshwater ecosystems processes such as nutrient loadings, harmful algae blooms, greenhouse gas emissions (methane), aquatic habitat shifts, etc. While this is an interesting topic, I feel that this paper is not suitable for Nature Geoscience for several reasons that I provide below.
We thank the reviewer for their careful reading of the manuscript and for raising these important points. We agree that the impacts of sediment heatwaves on biogeochemical and ecological processes remain subject to substantial uncertainty, and we have revised the Introduction, Discussion, and Conclusions to better reflect this, and softening the wording where appropriate.

We would like to clarify that the primary objective of this study is not to quantify the biogeochemical or ecological impacts of sediment heatwaves, but rather to provide the first global-scale assessment of the magnitude, duration, frequency, and future evolution of thermal extremes occurring within lake sediments. Establishing the spatiotemporal characteristics of these sediment heatwaves is a necessary first step toward evaluating their potential implications for freshwater ecosystems.

In response to the reviewer's comment, we have nevertheless strengthened the manuscript by providing quantitative context on the potential sensitivity of sediment methane production and sediment respiration to heatwaves – this analysis was also run to address the comment of reviewer 1, who specifically asked for quantitative estimates of the impact of lake sediment heatwaves on biogeochemical cycles. Our results are presented below.

These revisions clarify the scope of the study, temper causal claims, and position the results as a foundation for future work linking sediment thermal extremes to biogeochemical and ecological responses.

The occurrence of a heatwave was determined during the ice-free season using modelled temperature time series climatologies. Using an existing R package, a heatwave occurred when the daily temperature exceeded the 90th percentile. I found nothing wrong with this approach, however, the intensity of the heatwaves (historic and projected) are small, on the order of between 1 to 2 K (average), and the standard deviation was large (+/- 0.6 K). I don't find this result surprising, but I do feel that the motivations for the study as provided in the Introduction, and the points raised in the Discussion and Conclusions are far-reaching given such a small temperature change. In several of the references provided, I could not find any mention of how sediment temperature itself affects a process/organism. In others, the temperature increases required to have a significant impact on a stated processes were greater than the increase in sediment temperature associated with a heatwave reported here. For example, methane production (as many biochemical reactions) increases as Q10 functon(a 10 C rise for an increase in production), and depends not only on temperature but the liable carbon pool (how was that accounted for?). The release of phosphate, nitrogen and other nutrients in the sediment is also often a Q10 function. Many studies that incubate lake sediment find an increase once sediment temperatures increase by 10 or 20 C. These temperature increases that invoke a significant response are several times the magnitude of the sediment heatwaves reported here, and there are many other variables at play that are apparently not accounted for. Although there is little question that temperature does affect these processes provided in a few of the examples, in many instances, the cited literature

does not support these changes over the small range in sediment temperature increases during a heatwave. The justification of the study and the discussion points/conclusions should be framed in a realistic scope that is commensurate with the results, not to mention that there are a multitude of factors in addition to temperature that directly influence many of these processes.
We thank the reviewer for this detailed and constructive critique. We appreciate that the reviewer found nothing problematic with our approach for defining and calculating sediment heatwaves, as the development and application of a consistent framework to quantify sediment thermal extremes is the primary aim of this study.

We agree that it is essential to carefully align the scope and interpretation of the manuscript with both the magnitude of the simulated sediment heatwaves and the level of process representation. We first clarify that the 1–2 K values highlighted by the reviewer refer to changes in heatwave intensity relative to the historical period (i.e., future minus historical heatwave intensity), rather than the absolute intensity of future sediment heatwaves. In absolute terms, future sediment heatwave intensities typically reach ~2–4 K under high-emission scenarios. This distinction is now clearly stated in the revised Results and Discussion.

While individual sediment heatwave temperature anomalies could appear modest to, for example, the public, their potential biogeochemical relevance emerges when accounting for their persistence, recurrence, and spatial extent. Also, we note that many global climate impact assessments and policy frameworks focus on ecosystem responses to warming of approximately 1.5–2 °C (e.g., the basis of the Paris Agreement). In this context, evaluating the implications of spatially extensive and persistent sediment temperature anomalies of comparable magnitude is both relevant and necessary, even though laboratory incubation studies often explore larger temperature ranges to elucidate mechanistic responses.

In response to the reviewer's concerns and request for quantitative context, we now include an Arrhenius-type temperature sensitivity analysis to estimate the fractional change in sediment biogeochemical process rates attributable solely to heatwave-related warming. As mentioned above, this was also in response to reviewer 1.

Using empirically derived activation energies from global syntheses of lake sediment incubation studies (Gudasz et al., 2010; Yvon-Durocher et al., 2014; Jansen et al., 2022), we estimate that methane production in littoral sediments, where the majority of lake methane emissions originate, more than doubles during future sediment heatwaves. Specifically, methane production increases by factors of 2.06, 2.22, and 2.28 under SSP1–2.6, SSP3–7.0, and SSP5–8.5, respectively. When accounting for the fraction of the year affected by heatwaves, total methane production is estimated to increase by factors of 1.47, 1.92, and 2.01 across these scenarios. Similarly, pelagic sediment respiration increases by factors of 1.47–1.53 during heatwaves, with heatwave-integrated increases of 1.26–1.41 when duration is considered. These findings are described in the results section, with two supplementary tables describing the key results.

These calculations demonstrate that even moderate (2–4 K) but persistent sediment heatwaves can amplify temperature-dependent biogeochemical rates. We emphasize, however, that these estimates intentionally isolate the effect of temperature alone and do not account for additional controls such as sediment organic carbon availability, redox conditions, oxygen penetration depth, or microbial community composition. As such, they should be interpreted as indicative of potential sensitivity rather than predictions of realized methane or nutrient fluxes.

Importantly, the primary aim of this study remains the global quantification of sediment heatwaves as a previously unrecognized class of thermal extremes, rather than the explicit

simulation of biogeochemical fluxes. References to biogeochemical processes are now provided to illustrate why sediment heatwaves may matter in an Earth-system context, not to imply direct or universal causal impacts. We now explicitly state that robust projections of biogeochemical responses would require dedicated, process-resolving studies and should not be inferred directly from the present analysis.

We believe these revisions and additions address the reviewer's concerns while ensuring that the manuscript's motivation, discussion, and conclusions remain realistic, balanced, and commensurate with the scope of the study.

My second area of concern is related to the methodology. The paper relies on the use of the lake model (the ALBM), itself driven by other models (GCMs), with those driven by SSP models for climate change projections. Results are at times presented as one prediction against another using the same model, so results are expectedly good. Fig. S2 is an example of this; the comparison of lake surface or bottom temperature to littoral or pelagic sediment temperature is great (as expected) and was therefore used to the justify the use of bottom water temperature as a proxy for sediment temperature (line 349), but this is just a validation of the internal workings of the ALBM model. I fully appreciate the fact that the there are few direct measurements of sediment temperatures available and that the authors did offer a comparison using some measurement from 4 lakes (Fig. S15), however, those data are indeed very limited in geographic location and temporal coverage and therefore scaling from a handful of measurements to the entire globe over time scales of decades is a huge leap.
We thank the reviewer for raising this important point regarding model validation and the use of internally consistent model outputs. We agree that comparisons between different state variables produced by the same model (e.g., bottom water temperature versus sediment temperature within ALBM) primarily serve to demonstrate internal physical consistency rather than independent validation. We have revised the manuscript to clarify this distinction and to avoid overstating the implications of such comparisons.

The comparison shown in Fig. S2 is intended to illustrate the physical coupling between bottom water and upper sediment temperatures within the model framework and to motivate the mechanistic basis for using bottom water temperature as a first-order proxy for sediment thermal forcing in data-limited contexts. We now explicitly state that this analysis does not constitute an external validation of sediment temperatures, but rather demonstrates that the simulated sediment heatwaves are dynamically consistent with lake thermal structure as represented in ALBM.

We fully acknowledge the reviewer's concern regarding the scarcity of in situ sediment temperature observations suitable for large-scale validation. While observational constraints remain a fundamental limitation of global lake sediment studies, we have expanded our observational comparison by incorporating sediment temperature measurements from a few additional lakes, increasing the number of lakes used for external evaluation. These datasets provide independent, albeit still geographically limited, support for the model's ability to capture the timing and magnitude of seasonal and interannual sediment temperature variability.

Finally, we note that similar challenges in observational coverage are common to global assessments of terrestrial soil temperature extremes, permafrost dynamics, and ocean benthic warming, where physically based models are used to identify emergent large-scale patterns in the absence of comprehensive observations. We have added text to clarify that our results should be interpreted as first-order global estimates of sediment thermal extremes, intended to guide future observational and process-based studies rather than to replace them.

My final major concern relates to the spatial and temporal variation of sediment thermal and biological properties. Temperature alone cannot explain the likelihood of the occurrence of

many of the processes described in the Introduction, Discussion, and Conclusions, and the temperature response can vary regionally and within individual lakes. Methane release requires carbon-rich sediment; thermal stresses vary with species and location. Sediment temperature depends not only on adjacent water temperature (and all the variables that control that), but the sediment's thermal conductivity/heat capacity. Horizontal advection of heat along shorelines with shallow waters could also be important and not represented by a 1-D model. I saw no mention of which values(s) were used, how there were determined, or the sensitivity of sediment temperature to a realistic range in sediment thermal properties. Most lakes, even large, deep ones, have many shallow areas in bays and river inlets, and I wondered how these are resolved in within a 0.5 x 0.5 degree grid.
We thank the reviewer for raising these important points regarding spatial heterogeneity, sediment properties, and model structure. We fully agree that temperature alone cannot explain the likelihood or magnitude of many biogeochemical and ecological processes, and that sediment thermal and biological responses can vary substantially within and among lakes depending on local conditions. Indeed, as mentioned above, this was not the intention of our study.

Our study does not seek to represent this full complexity. Rather, it focuses on quantifying the physical characteristics of sediment thermal extremes at the global scale, using a process-based but necessarily simplified modelling framework. We have revised the manuscript to more clearly acknowledge these limitations and to avoid implying that sediment heatwaves alone determine biogeochemical or ecological outcomes.

With respect to sediment thermal properties, the Advanced Lake Biogeochemistry Model (ALBM) follows the ISIMIP3b lake-sector protocol and applies globally consistent sediment thermal conductivity and heat capacity values derived from established literature (e.g., Fang and Stefan, 1998; Tan et al., 2015; Tan et al., 2024). This approach ensures internal consistency across the representative lakes simulated worldwide, but does not resolve lake-specific or sub-lake-scale variability in sediment composition. We now explicitly state these assumptions in the Methods section and note that sensitivity of sediment temperatures to local sediment properties represents an important area for future work.

Regarding spatial heterogeneity, ALBM represents lakes using a single vertical water column coupled to multiple sediment columns that span different fractions of the lake area and contact with different depths of lake water (see Figure S1 in Tan et al. (2024) and our response to reviewer 1). The shape of the water column is defined by the lake-specific bathymetry that is derived by the ISIMIP3b lake-sector protocol. This model architecture allows us to distinguish between littoral and pelagic sediments in this study, which captures first-order contrasts between shallow nearshore and deep basin environments. While this approach does not resolve fine-scale features such as individual bays, river inlets, or horizontal heat advection along shorelines (which are most important for large lakes, not the vast number of small lakes worldwide, and most represented in this study), it provides a physically consistent approximation of spatial variability that is appropriate for global-scale analyses. Note that a global study capturing the 3-dimensional complexity of lakes would not be possible (by anyone) in the foreseeable future.

We agree that processes such as horizontal heat transport in shallow regions, intermittent sediment exposure, and locally elevated organic carbon availability may further modulate sediment temperatures and biogeochemical responses. These processes are not explicitly represented in the current 1-D framework (required for global scale studies) and would require high-resolution, lake-specific models and observations. We have added text in the Discussion to highlight these limitations and to emphasize that our results should be interpreted as representing large-scale, first-order patterns rather than local extremes.

Finally, we reiterate that the primary aim of this study is not to predict site-specific biogeochemical fluxes or biological responses, but to introduce and globally quantify sediment heatwaves as a previously unrecognized class of thermal extremes. By identifying their spatial distribution, timing, and projected changes under climate warming, our results provide a necessary physical foundation for future observational and modelling studies that can explicitly address the roles of sediment properties, ecosystem state, and local hydrodynamics.

We believe that these clarifications appropriately contextualize the limitations of the modelling approach while maintaining the scientific value and novelty of the global-scale analysis.

As mentioned above, we have softened the wording throughout the manuscript when describing the possible implications of the physical changes that we report. When the biogeochemical responses are discussed, we mostly limit the discussion to methane production and sediment respiration, which we now provide some first-order estimates.

For these reasons, I feel that this paper is not appropriate for Nature Geoscience and is much better suited for a model-focused journal such as JAMES or Geosci Model Dev. since this is essentially a modelling paper with results and conclusions that are nearly entirely model-based.

We respectfully disagree with the reviewer's assessment that this study is primarily a model-development exercise and therefore more suitable for a model-focused journal. While our analysis relies on a process-based lake model, the model itself is not the focus of the manuscript. Rather, it is used as a tool to address a previously unexplored geophysical phenomenon: the emergence, characteristics, and future intensification of lake sediment heatwaves at the global scale.

The primary novelty of this work lies in (i) introducing sediment heatwaves as a new class of climate extremes, (ii) demonstrating their distinct physical behaviour relative to surface and subsurface lake heatwaves, (iii) identifying their seasonality, persistence, and lagged response to surface conditions, and (iv) quantifying how these extremes are projected to change under different climate scenarios. These are fundamentally geoscientific questions directly aligned with the scope of *Nature Geoscience*, which regularly publishes model-based studies when they reveal new Earth system behaviours or emergent climate risks.

Global-scale observational datasets of lake sediment temperature do not currently exist, making a modelling approach the only feasible pathway to identify first-order patterns, assess physical mechanisms, and inform future observational and experimental efforts. This situation is analogous to early global assessments of marine heatwaves, permafrost thaw, or subsurface ocean warming, which were likewise model-driven at the time of their introduction and have since become central to climate impact science.

Importantly, this manuscript does not present or validate a new model, nor does it focus on model architecture, parameter optimization, or numerical performance - hallmarks of model development journals such as JAMES or Geoscientific Model Development. Instead, it uses an established, community model within the ISIMIP framework to address a broad, interdisciplinary climate question with clear implications for freshwater systems, biogeochemical cycling, and ecosystem resilience.

We also note that three independent reviewers evaluated the manuscript positively and recognized its conceptual novelty and relevance. In response to the concerns raised here, we have substantially revised the Introduction and Discussion to better align the framing, scope, and implications with the physical results presented, while explicitly acknowledging uncertainties and limitations.

We therefore believe that the manuscript is well suited to *Nature Geoscience* and contributes new, policy-relevant insight into how subsurface components of the Earth system respond to climate extremes.

A note on the References:

I found that several of the cited papers I could not find the relevant information as referenced in the text. For example:

Line 335: 'For example, ALBM has previously been used to simulate lake temperature, as well as changes to stratification and mixing cycles in lakes, and has been shown to accurately reproduce temporal variations in bottom water AND sediment temperatures (Tan et al., 2015; Woolway et al., 2021b; Golub et al., 2022).'
Thank you for casting a careful eye on the references. Indeed, most of these references demonstrated the ability of ALBM in simulating the water column thermal dynamics.

However, we are somewhat confused by the contradiction in the reviewers comment here and below. Below they state that Tan et al., (2015) showed a comparison between measured and modelled sediment reported for one winter period in Goldstream Lake (Alaska). Thus, the reference and statement provided above is correct. We have now slightly modified this text in the paper – but please let us know if we misunderstood. Note that the references reported above cover individual lake thermal properties (i.e., each paper does not look at all variables).

Tan et al. 2015. This paper deals with Arctic lakes/methane, and list another 2015 paper that does deal with temperature validation as 'at press' (the proper citation is missing). The JAMES paper by Tan, Zhuang, and Anthony (2015) (also on the topic of methane release from pan-Arctic lakes). In this paper I see 1 lake (Goldstream Lake, Alaska) with a comparison between measured and modelled sediment reported for one winter period (Sept/2008 – May/2009) when temperatures were largely in the 4-5 C range.
Thank you for this comment. The relevant figure from Tan et al., (2015) is shown below. Sediment temperatures (shown in blue) vary from approx. 4 C to 8 C.

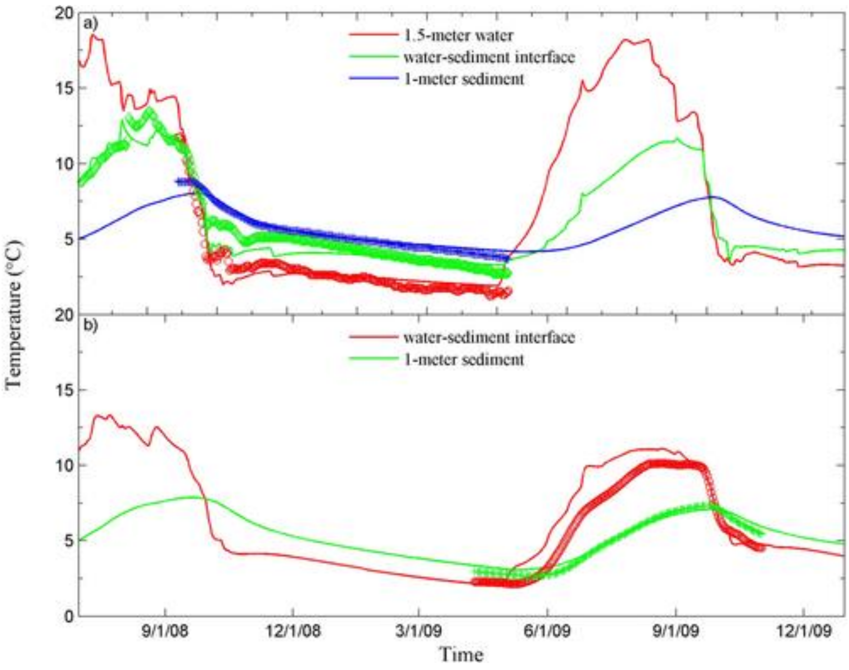

Fig. R2. Comparison of the simulated (solid lines) and observed (symbols) temperatures at Goldstream Lake, Alaska (For the 2.3 m deep nonthermokarst lake center, temperatures were

recorded from 3 July 2008 to 4 May 2009; For the 1.5 m deep thermokarst margin, temperatures were recorded from 10 April 2009 to 11 November 2009). From Tan et al., (2015).

In the methods section, we now add the following text:

In addition to these datasets, previous validation of ALBM against sediment temperature observations has been conducted for Goldstream Lake, a thermokarst lake in Alaska (Tan et al., 2015). Modelled temperatures were compared with measurements at both the lake center and margin, including the water–sediment interface and sediment layers up to 1 m depth. The model reproduced observed thermal dynamics with mean errors generally below 0.6 °C in the water column and below 0.3 °C in sediments, with slightly larger discrepancies at lake margins where horizontal heat exchange with surrounding permafrost moderates warming. The simulations also captured the observed seasonal progression of thermal maxima, which occurred first in midwater during July, then at the lake bottom in August, and finally within surface sediments in September, reflecting downward heat transport from the atmosphere. These results demonstrate ALBM's ability to represent coupled water–sediment heat transfer processes across both central and marginal lake environments, providing confidence for its application to sediment temperature simulations in diverse lake systems.

Woolway et al 2021b - I could not find mention of sediment temperatures.
Yes, this study focused on water column temperature. Our study is the first to look at lake sediment temperatures globally.

Golub et al 2022 - I could not find mention of validation/calibration of sediment temperatures.
Yes, this study focused on water column temperature. Our study is the first to look at lake sediment temperatures globally.

Reviewer #3:
The authors did an interesting study investigating lake sediment heatwaves under global warming, which is a large scale study of great impact. Reviewing the whole manuscript, I found several points that might be able to improve:

(1) Comparing heatwaves between sediment and lake surface: How about comparing with subsurface lake heatwaves? The authors' study "Subsurface heatwaves in lakes", published at NCC, investigated subsurface lake heatwaves. In my understanding, lake bottom water interacts directly with sediment, thus, it is reasonable to compare heatwaves in these layers.
We thank the reviewer for this suggestion. We agree that bottom water temperature is closely linked to sediment thermal dynamics, and we included comparisons between sediment and bottom water heatwaves in our analysis (see Fig. S2). We now clarify this in the text and emphasize that pelagic sediment heatwaves are closely associated with bottom water heatwaves, consistent with previous studies on subsurface heatwaves. This provides a physically consistent rationale for our sediment-focused analysis while linking it to established subsurface heatwave metrics.

The main reason for comparing lake sediment heatwaves to lake surface heatwaves was to see if these sediment extremes could be anticipated based on surface water temperature data, which is widely available via satellite Earth Observation. If these were highly correlated, then one gain reasonable information from surface observations on extremes in the sediment (pelagic or littoral). But, in response to the reviewer comment, we now also compare these sediment heatwave characteristics to bottom water heatwaves (see Fig. S6). The results are presented as supplementary figure and discussed in the main text.

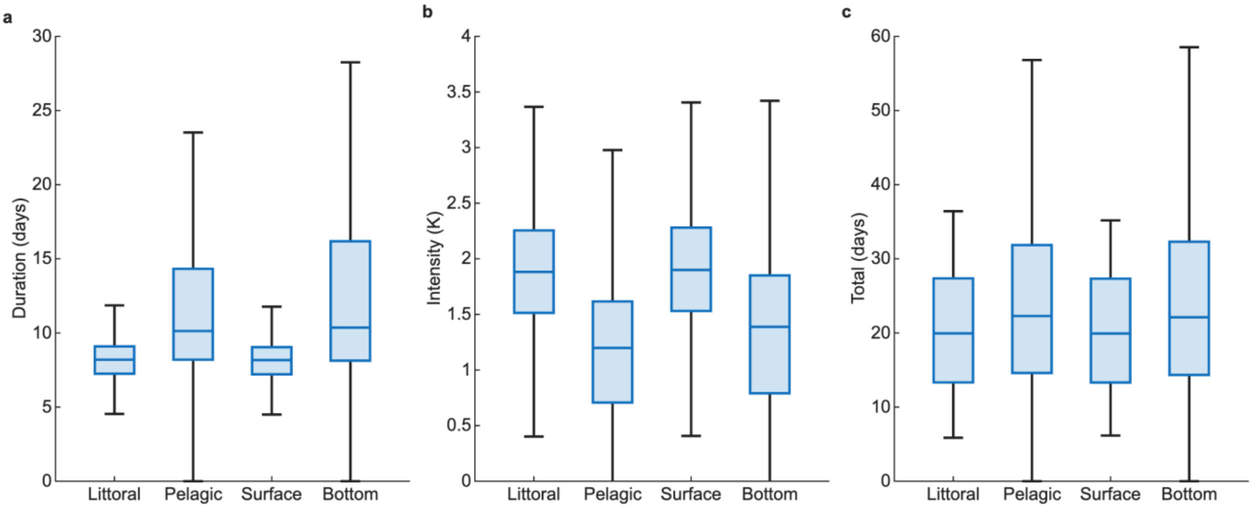

Figure S6. Comparison between littoral, pelagic, surface and bottom water heatwaves.

(2) Why these five GCMs are selected?
We thank the reviewer for this question. The five GCMs used in this study (GFDL-ESM4, IPSL-CM6A-LR, MPI-ESM1-2-HR, MRI-ESM2-0, and UKESM1-0-LL) were selected because they are part of the ISIMIP3b lake-sector protocol and provide the bias-corrected, high-temporal-resolution (daily) meteorological forcing required to drive ALBM consistently across historical and future periods. Other GCMs available in CMIP6 are not bias-corrected, which is important for small lakes i.e., to be representative of over-lake conditions.

These models are widely used in global impact assessments across multiple Earth-system sectors, including lakes, hydrology, ecosystems, and biogeochemistry, and have undergone extensive evaluation within the ISIMIP framework. Importantly, this ensemble spans a broad range of equilibrium climate sensitivities and transient warming responses, allowing us to capture inter-model uncertainty in projected lake and sediment thermal regimes.

Using this protocol-based, multi-model ensemble ensures methodological consistency with other global lake studies and enables robust estimation of ensemble-mean responses and uncertainty ranges, rather than reliance on any single climate model.

(3) Why omit SSP245 for scenario setup?
We thank the reviewer for this question. SSP2-4.5 was omitted because the bias-corrected daily climate forcing required to drive ALBM is not currently available for this scenario within the ISIMIP3b lake-sector protocol. At present, ISIMIP3b provides consistent, bias-corrected outputs for SSP1-2.6, SSP3-7.0, and SSP5-8.5 only.

Our scenario selection therefore follows the ISIMIP3b protocol to ensure methodological consistency with other global-scale lake modelling studies and to enable robust multi-model ensemble analysis. The three scenarios used span a wide range of plausible future forcing trajectories - from strong mitigation to high-emissions - thereby capturing the key contrasts relevant for assessing future changes in sediment heatwaves.

(4) For model performance, it is better to report the RMSE value as well, a widely used metric to evaluate model performance.
We agree that reporting RMSE is informative. We have added RMSE values for surface and sediment temperature validation against available observational datasets. These metrics confirm that ALBM reproduces observed temperature dynamics with reasonable accuracy, consistent with previous validations.

(5) Some figures shall be improved in quality and presentation. The detailed comments can be found in the attached file.
We thank the reviewer for this suggestion. We have improved figure resolution, readability, and consistency of axes, labels, and colour scales throughout the manuscript and supplementary information to enhance clarity and presentation quality. Our changes directly address the suggestions made in the annotated file submitted by the reviewer.

Reviewer #4
The authors present the first global-scale projection and analysis of lake sediment heatwaves, including both historical and future periods, using an extensive dataset. The Introduction and Results sections are well written, and the figures are generally clear and appealing. My comments mainly concern the Methods/Discussion sections, along with several minor points detailed below.
We thank the reviewer for their positive evaluation of our manuscript and for recognizing the novelty of providing the first global-scale analysis and projection of lake sediment heatwaves. We are pleased that the reviewer found the Introduction, Results, and figures to be clear and well presented. Below, we address the reviewer's comments concerning the Methods and Discussion sections, and we have revised the manuscript accordingly to improve clarity, transparency, and interpretation.

Methodological Comments
Line 300
Is the bathymetry lake-specific, or is a representative bathymetry used for all lakes? Clarification is needed here. Bathymetry can influence the spatial discretization of the model grid, the definition of littoral versus pelagic zones, and lake stratification processes. If a representative bathymetry is applied to all lakes, this choice should be made explicit and ideally illustrated in the Supporting Information for transparency.
We thank the reviewer for this important clarification request. In the ISIMIP3b lake sector, each 0.5° × 0.5° grid cell the bathymetry is derived from global datasets and scaled to represent the aggregated lake area within that grid. Thus, bathymetry is grid-cell specific. We have clarified this explicitly in the Methods section and added a brief description and illustration of the lake depth in the Supporting Information for transparency (Fig. S19).

Lines 318–323
The model grid configuration is not sufficiently clear. A schematic diagram illustrating the lake and sediment discretization would be helpful. I consulted the original ALBM model paper, where I understand that N sediment columns can be used — could the authors explain the choice of using four columns specifically?
We thank the reviewer for this helpful suggestion. We agree that a schematic would improve clarity. Such schematic diagram is provided in fig S1 of Tan et al., (2024) – see below. We now specifically refer to this figure in the text. However, as it has already been published elsewhere, we have not included it in the current manuscript. If a reworked version of this figure is needed, we are happy to provide one. But, at present, we hope that referring to Fig. S1 of Tan et al., 2024 is sufficient.

In ALBM, each lake is represented by a single vertically resolved water column coupled to multiple laterally distributed sediment columns located along an idealized depth gradient. In this study, we use four sediment columns, consistent with the standard ISIMIP3b lake-sector configuration. These columns represent a continuum from nearshore (littoral) to deep (pelagic) sediments, with two intermediate depth zones.

Each sediment column is vertically discretized into 40 layers, resolving thermal dynamics within the upper sediment, including the biologically and biogeochemically active upper 1 m used in the heatwave analysis. While ALBM allows for a flexible number of sediment columns, the four-column configuration reflects a trade-off between physical realism and computational feasibility and has been adopted to enable consistent global-scale simulations across tens of thousands of lakes within the ISIMIP3b framework.

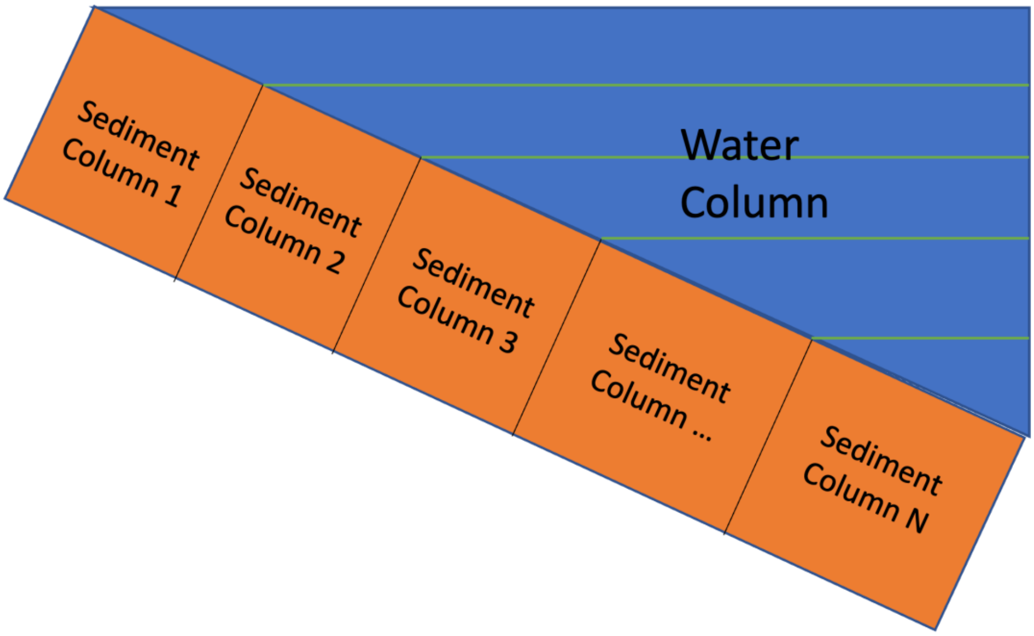

Figure R1. Schematic representation of the ALBM lake–sediment discretization used in this study. Each lake is represented by a single vertically resolved water column coupled to four laterally distributed sediment columns spanning a nearshore (littoral) to deep (pelagic) depth gradient. Each sediment column is vertically discretized into 40 layers, resolving thermal dynamics in the upper sediment. Sediment heatwaves are evaluated within the upper 1 m of each column.

Additionally, the definition of littoral and pelagic zones requires clarification. Is the same spatial definition applied for all lakes regardless of depth? Are littoral sediments assumed to be alternately exposed to the atmosphere and submerged (i.e., forced alternately by atmospheric conditions and water temperature), or are they always submerged?
We thank the reviewer for raising this important point and agree that the definitions of littoral and pelagic sediment zones require clarification.

In this study, the spatial definition of littoral and pelagic sediments follows the standard ALBM/ISIMIP3b lake-sector configuration and is applied consistently across all lakes, independent of absolute lake depth. The four sediment columns represent an idealized depth gradient from nearshore (littoral) to deep (pelagic) sediments, rather than site-specific bathymetry. As such, the littoral column should be interpreted as representing relatively shallow, nearshore sediments compared to the pelagic column, but not as a fixed depth threshold that is uniform across lakes.

Importantly, lake water levels are held constant in the ISIMIP3b protocol, and processes associated with lake-level fluctuations are not represented. Consequently, all sediment columns, including the littoral sediment column, are assumed to remain permanently submerged throughout the simulation period (now also described in the limitations section – see Methods). Littoral sediments are therefore forced exclusively by overlying water temperatures rather than alternating between atmospheric and aquatic forcing.

We have clarified these assumptions in the manuscript and now explicitly state that the littoral–pelagic distinction in this study reflects relative position along a depth gradient within the lake model, rather than dynamic exposure or shoreline migration.

Related to this, was seasonal lake-surface-area variability (wet/dry periods) considered?

We thank the reviewer for this important clarification request. As explained above, the ISIMIP3b lake sector protocol does not consider seasonal lake-surface-area variability. We have clarified this explicitly in the Methods section.

Regarding vertical discretization, stating the use of “40 sediment layers and 50 water layers” does not adequately convey the true spatial resolution unless layer thickness is also specified. Given that results and validation are presented at 0.5-m or 1-m sediment depths, is this resolution achievable with the applied discretization, particularly for deep lakes where layer thickness may exceed these values?
We thank the reviewer for this important clarification request. The ALBM model uses variable layer thickness to represent water and sediment columns. Specifically, the layer thickness of water and sediment columns increases exponentially from the surface to the bottom. The thickness of the first water and sediment layers is always roughly 0.1 m for all lakes. The first 1-m water and sediment depths are usually represented by at least five layers. This discretization scheme ensures that the water and sediment layers where intense thermal dynamics occur can be resolved properly. As a result, the applied discretization can achieve the resolution needed for the analysis and validation at 0.5-m or 1-m sediment depths. We have clarified this explicitly in the Methods section. We also note that most of the representative lakes in ISIMIP3b are not very deep (see Fig. S19).

This also raises the question of why 1 m depth is chosen for the main analyses. Is sediment at 1 m depth still considered representative of sediment surface temperature?
We thank the reviewer for this important clarification request. The 1 m depth is chosen because the sediment within the top 1 m is where most biological and biogeochemical processes occur – please note that this is the average of the top 1m. To be comparable across lakes, a 1m sediment layer was selected. This is also similar to the definition often used of lake surface water temperature, where observations are averages throughout the top 1m. We also note that for the very few validation data available, these were restricted to the upper sediment layer

Line 363
Validation against bottom-water temperature may demonstrate model skill for predicting pelagic surface sediment temperature, but it does not necessarily test performance for littoral sediments — particularly if these sediments experience alternating exposure and submergence. If littoral sediments are assumed to remain permanently submerged, this should be clarified explicitly in the Methods section.
We thank the reviewer for this important clarification. We agree that validation against bottom-water temperature primarily assesses model performance for pelagic sediment thermal forcing and does not explicitly test littoral sediment temperatures, particularly in systems where littoral zones may experience periodic exposure. However, in the ISIMIP3b lake-sector simulations, littoral sediments are assumed to remain permanently submerged, and processes related to sediment exposure or rewetting are not represented. We have clarified this assumption explicitly in the Methods section and have revised the validation discussion to avoid overstating model skill for littoral sediment temperatures.

Lines 393–394
It appears that future heatwaves may be defined relative to the present baseline (1991–2020). If this interpretation is correct, it would explain why heatwave conditions persist year-round in some future scenarios. However, clarification is needed here. If present-day heatwaves are defined relative to a present baseline, it seems equally reasonable that future heatwaves could be defined relative to a future baseline as well. The authors should clarify which baseline is used for future heatwave identification and explain the rationale for this choice, as this definition underpins all subsequent analyses.
We thank the reviewer for highlighting this important issue, which is actively debated in the heatwave literature. In this study, future sediment heatwaves are identified using a fixed

historical baseline derived from the reference period (1991–2020). This choice follows the conventional definition of heatwaves in atmospheric and marine systems and is consistent with recent assessments emphasizing that fixed-baseline approaches are essential for quantifying the increasing frequency, duration, and intensity of thermal extremes under climate change (Hobday et al., 2016).

Using a shifting baseline addresses a different research question focused on relative variability around a warming mean state, but it can artificially mask the emergence of extreme conditions and decouple physical forcing from biological and biogeochemical responses that operate on different timescales. A fixed-baseline definition aligns with World Meteorological Organization recommendations and is widely adopted in climate assessments to ensure consistency in communicating evolving climate extremes across disciplines and to stakeholders. We have clarified this definition and rationale explicitly in the Methods section.

The choice of a fixed historical baseline is critical for interpreting future sediment heatwaves. Fixed-baseline definitions capture the increasing exposure of benthic environments to extreme temperatures relative to present-day conditions and are consistent with evidence linking marine and freshwater heatwaves to increasingly frequent and severe ecological impacts. In contrast, shifting-baseline approaches may obscure long-term changes in absolute thermal stress and create the misleading impression that heatwave risk remains stationary despite continued warming. Maintaining a fixed baseline also ensures consistency with established climate assessments and facilitates comparison across atmospheric, marine, and freshwater systems.

We have also made a note of this in the discussion section.

Line 441
Further details on the cross-correlation function would help readers understand precisely how time lags are calculated.
We thank the reviewer for this suggestion. We have expanded the Methods section to provide additional details on the cross-correlation analysis, including how time lags were computed and interpreted. These clarifications specify the time series used, the range of lags considered, and the criterion used to identify the dominant lag between surface and sediment heatwaves.

Minor Textual Issues
Lines 152–157 and 160–164
These sections appear to be repetitive.
Corrected. Thank you

Forcing Data & Interpretation
Line 172
The projected future results presumably depend strongly — or even entirely — on the meteorological forcing data, yet no information on these forcings is provided. At minimum, summary descriptions of projected air temperature, solar radiation, or other relevant drivers should be included to help readers understand the physical reasons behind the projected changes in sediment heatwaves and to improve transparency.
Thank you for this suggestion. We have now added a figure showing future changes in air temperature, solar radiation and wind speed globally (Fig. S12).

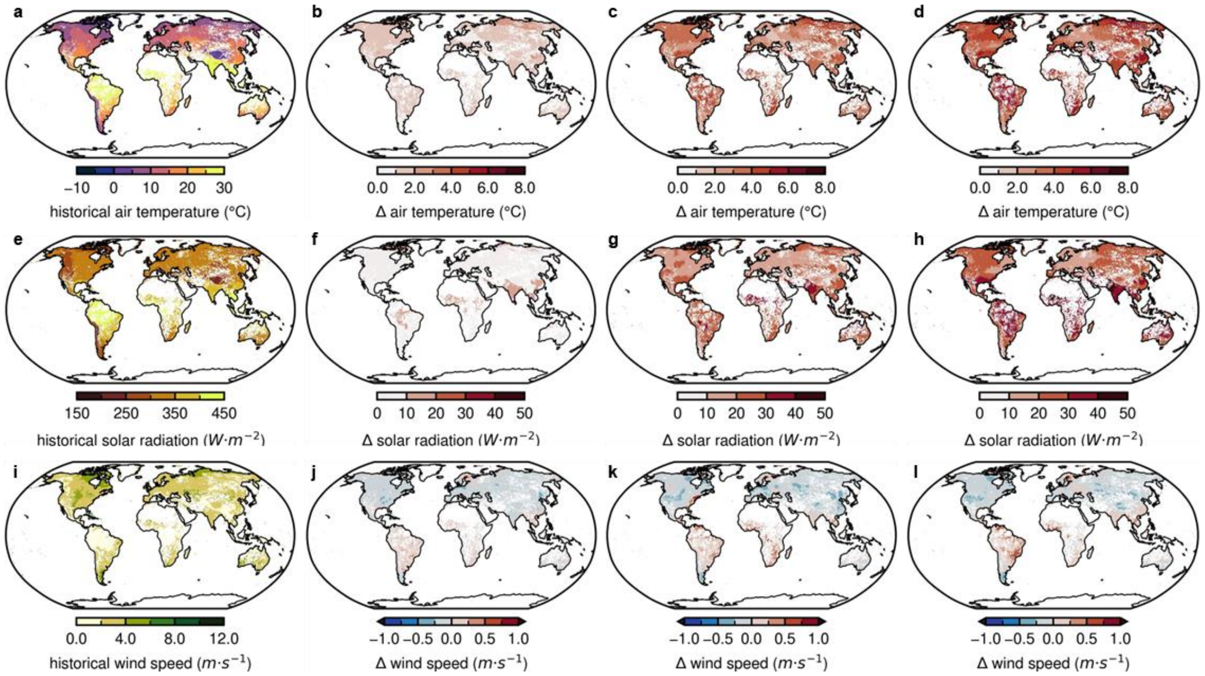

Figure S12 | Historical and future patterns in atmospheric forcing. Shown are global variations in historic and future changes in (a-d) air temperature, (e-h) solar radiation, and (i-l) wind speed. Historical patterns are shown in panels a, e and i. Future changes are shown for SSP126 (b, f, j), SSP370 (c, g, k) and SSP585 (d, h, l). Future changes refer to differences by the end of the 21st century relative to historic conditions.

Line 221
Following the previous point, the Discussion focuses primarily on the ecological and biogeochemical implications of future sediment heatwaves. Additional discussion of the modeling results themselves would be valuable — including discussion of the projected forcing trends, model uncertainty and sensitivity, and justification of key methodological choices.
Thank you. We have now updated the discussion (and methods) to include more details of the modelling results, the forcing trends, model uncertainty, and some justification of key methodological choices.

Comments on Figures
• Figure 1h — The small panel and the global map are not well aligned with each other (e.g., relative to panels g and i).
We have now reproduced this figure and moved the small panels (boxplot) to a separate figure. Thank you for bringing this to our attention.

• Figure S2a — Surface water temperature appears consistently higher than littoral sediment temperature, while bottom-water temperature appears lower than pelagic sediment temperature. This is interesting and merits further explanation if not already discussed.
We attribute this pattern to (i) that many of the studied lakes are shallow, and thus solar radiation can penetrate to the sediment, leading to excess warming; and (ii) the higher thermal inertia of sediments, which act as a heat reservoir. During spring and summer, sediments accumulate heat, which is retained through autumn and winter, even as bottom waters cool rapidly due to heat loss to the atmosphere. This results in a lag where sediment temperatures remain higher than bottom-water temperatures. In contrast, surface water in littoral regions responds more quickly to atmospheric forcing. We have added a brief note discussing this in the revised manuscript.

Note that this effect has also been reported in other studies. Notably, the study of Tan et al., (2015) showed that pelagic sediment temperatures can be warmer than the water above due to thermal inertia effects.

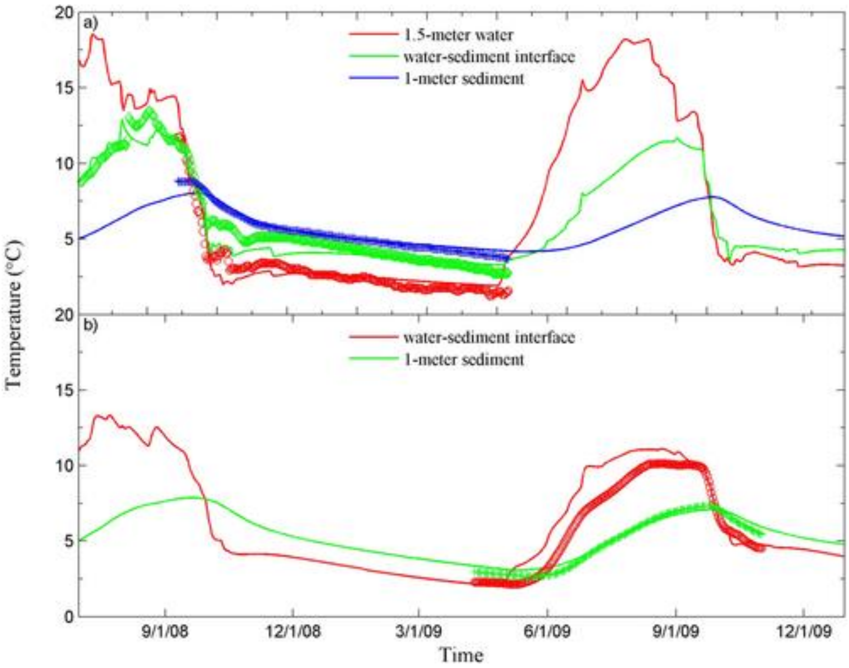

Fig. R2. Comparison of the simulated (solid lines) and observed (symbols) temperatures at Goldstream Lake, Alaska (For the 2.3 m deep nonthermokarst lake center, temperatures were recorded from 3 July 2008 to 4 May 2009; For the 1.5 m deep thermokarst margin, temperatures were recorded from 10 April 2009 to 11 November 2009). From Tan et al., (2015).

• Figure S3 — Only panel (d) includes “Scatter” in the title, whereas panels (b) and (c) do not. For consistency, it would be preferable to standardize the panel titles.
Thank you. This has now been corrected.

• Figure S15c — In the Lake Mendota panel, the legend entry “insitu (lit)” is missing the left parenthesis.
Corrected.

Additionally, model uncertainty, sensitivity, and any systematic deviations observed during validation should be discussed in the Discussion section.
Noted. Thank you. We have now added these discussion points.

Reviewer #1 (Remarks to the Author):
I think the authors have addressed all the points I have raised. I suggest the manuscript can be accepted after address some minor questions all the reviewers raised.
We thank the reviewer for their careful reassessment of our manuscript and for their positive feedback. We appreciate the reviewer's comments and suggestions, and we have carefully addressed the minor points raised by all reviewers in the revised manuscript.

1.The statements such as "first global-scale assessment", "first systematic assessment" have been emphasized several times at different parts. I think this statement in the abstract is enough.
Thank you for pointing this out. We have changed the text accordingly.

2.Significance test of the difference in Fig. S6 should be added like in Fig. S4.
Yes, of course. We have now modified the figure accordingly. Note that the figure numbers have now changed.

3.The lake model, ALBM is driven by outputs from five GCMs. Please add the discussion on the lake sediment heatwave difference related to inputs or the climate model uncertainty.
We thank the reviewer for this important suggestion. We have expanded the *Further considerations* section (see Methods) to explicitly discuss uncertainty associated with the use of multiple GCM forcings. The revised text now clarifies how differences among climate models in air temperature trends, radiation balance, wind forcing, and ice phenology influence the magnitude and timing of simulated lake warming, and therefore the projected intensity and duration of sediment heatwaves. We further clarify that while inter-model differences contribute to uncertainty in the magnitude and regional expression of change, all climate forcings consistently project increases in sediment heatwave frequency and duration, indicating that the emergence of sediment heatwaves is a robust response to climate warming. This addition is intended to better contextualize the projections within the broader framework of climate-model uncertainty.

Reviewer #2 (Remarks to the Author):

I thank the authors for their careful revisions and respectful responses to my concerns. I find the paper is much improved, and is now suitable for publication.

Thank you very much for your careful review of our manuscript. We sincerely appreciate your constructive comments and thoughtful suggestions, which have helped us improve the quality and clarity of the paper. We are grateful for your positive assessment of the revised version.

Reviewer #3 (Remarks to the Author):
The authors have addressed all my concerns. I don't have any further comments. If the other reviewers are satisfied with the revision, the paper is acceptable in my opinion.
We sincerely thank the reviewer for their careful evaluation of our revised manuscript and for confirming that their concerns have been addressed. We greatly appreciate the reviewer’s time and constructive feedback, which have helped improve the quality of the paper.

Reviewer #4 (Remarks to the Author):
The authors answered my questions quite clearly and added the required explanations in the updated manuscript, especially regarding the reasons for their methodological choices and the discussion of limitations and uncertainties in their results. The newly added section provides a clear description of the Arrhenius-based framework used to quantify heatwave impacts on sediment biogeochemical rates. However, several points require clarification or refinement to ensure mathematical correctness, internal consistency, and appropriate interpretation.
We would like to thank the referee for their careful and considerate review of the newly added section. We fully agree with their comments and have made the necessary changes to the manuscript.

1. Clarification of the Arrhenius formulation (Eq. 1)
Equation (1) represents a normalized Arrhenius-type temperature response using an empirical activation energy $E_a^{'}$ expressed in eV, together with Boltzmann's constant k_B. This formulation is appropriate and internally consistent. However, I think $E_a^{'}$ should be described as the slope of the relationship between $\ln J$ (or equivalently $\ln(J/J(T_c))$) and 1/T, rather than the slope of the logarithm of a centered rate difference ($J(T_c)-J(T)$). The Arrhenius relationship is linear in the logarithm of the rate (or rate ratio), not in the logarithm of a rate difference. Please correct me if my understanding is wrong.
We applied the equality ln(a) / ln(b) = ln(a-b) to format the left-hand side of the equation to match the right-hand side. The same linear relationship between logarithms of the centered values of J and T can be seen, for example, in Figure 1 of Yvon-Durocher et al., 2014 (https://www.nature.com/articles/nature13164), which we reference in the Methods. A complete derivation of Eq. 1, including the standardization step, can be found in the supplementary material of Yvon-Durocher et al., 2012 (https://www.nature.com/articles/nature11205).

In addition, the authors state that T_c is 16.8 °C and 13.4 °C in the littoral and pelagic zones, respectively. It should be noted whether these two values are derived from model output (if that is the case). It should also be explicitly stated that both T and T_c are converted to Kelvin prior to evaluation. While this may be implicit, stating it clearly would avoid ambiguity.
We converted the Tc values from °C to K to avoid ambiguity and stipulated that these values are indeed derived from model output.

2. Definition of heatwave temperature forcing and its implications
The manuscript defines heatwave intensity as the mean temperature anomaly relative to the seasonal climatology during heatwave events, calculated exclusively over the ice-free season. In Eq. (1), the temperature used to compute the Arrhenius response is defined as: $T=T_{\text{"historic ice-free mean"}} + \overline{(I_{\text{"HW"}})}$. This represents a single "effective heatwave temperature" constructed from a long-term mean plus an event-based anomaly. While this is a pragmatic simplification, it should be emphasized that this approach estimates the Arrhenius response evaluated at an adjusted mean temperature, rather than the true mean reaction rate under temporally varying temperatures during heatwaves.
Because Arrhenius functions are nonlinear, the rate evaluated at a mean temperature generally differs from the mean of rates evaluated over fluctuating temperatures. As such, this approach provides an effective or first-order estimate of heatwave impacts, but may

underestimate the contribution of peak temperatures during heatwaves. Explicitly acknowledging this approximation would strengthen the methodological transparency. Discussion is, of course, welcome if my understanding is not correct.

We agree with the Reviewer, and added the following to the Methods: "Our estimates of *J(T)* and J_{tot} are likely conservative because the use of long-term mean temperatures omits temperature peaks during heatwaves which would have a disproportionate impact on methanogenesis and respiration due to the non-linear nature of the Arrhenius-type temperature relation (Eq. 1),"

3. Consistency between heatwave intensity and duration weighting (Eq. 2)

Equation (2) computes the annual mean rate multiplier as a duration-weighted average, assuming:

a normalized rate of 1 during non-heatwave days, and

a rate of J(T) during heatwave days.

Given that heatwave intensity is defined as a mean anomaly conditional on heatwave events (rather than an annual mean anomaly), this formulation is mathematically consistent, provided that:

(I_"HW") ̅ is calculated only over heatwave days, and

t_"hw,tot" represents the total number of heatwave days in the year.

It would nevertheless be helpful to explicitly state this conditionality to avoid confusion and to clarify that intensity and duration are not double-counted.

We agree, and have clarified the text in the Methods as follows: "Because T in J(T) reflects the mean temperature during heatwave periods only rather than the mean annual temperature, heatwave intensity and duration are not double-counted in Eq. 2."

In addition, the assumption that non-heatwave days have a fixed normalized rate of 1 implies that broader background warming outside heatwaves is not considered; this assumption should be stated explicitly and briefly justified.

We have clarified our assumption in the Methods as follows: "The assumption of a rate of 1 outside the heatwave periods ensures that we only assess the direct impact of heatwaves rather than the broader impact of climate warming."

4. Interpretation of "rates" as relative multipliers

The computed quantities J(T)and J_"tot" represent dimensionless relative rate multipliers rather than absolute methanogenesis or respiration rates. This distinction is explained later in the text, but to avoid misinterpretation, it may be clearer to consistently refer to these quantities as "relative rates," "rate multipliers," or "fractional temperature effects," rather than simply "rates."

We have clarified the definition throughout the manuscript per the Reviewer's suggestion.

5. Choice and uncertainty of activation energy values

The use of globally synthesized E_a^'values for methanogenesis and organic carbon mineralization is appropriate for a global-scale analysis. However, given the known variability in apparent activation energies across substrates, redox conditions, and microbial pathways, a brief discussion of uncertainty or sensitivity to E_a^'would strengthen the robustness of the conclusions. Even a short acknowledgment of this limitation would be sufficient.

This is an important observation, and we have addressed this in quite some detail in a previous publication on (relative) methanogenesis rates (Jansen et al., 2022, DOI: 10.1111/gcb.16298), which includes a sensitivity analysis. Rather than repeating that discussion in its entirety, we opted to add a brief summary in the Methods with appropriate references to the original literature.

"We assumed here that the Ea' values (Eq. 1) will be constant over time, while laboratory and mesocosm experiments suggest Ea' might change due to synergistic temperature effects, such as adaptation of microbial communities, enhanced primary production or eutrophication (Borrel et al., 2011, Davidson et al., 2018, Zhu et al., 2020). However, the wide variety of natural aquatic ecosystems sampled to obtain the mean Ea' values in Yvon-Durocher et al. (2014) and Gudasz et al. (2010) have widely different properties and warming histories yet still converge to very similar temperature relations. In a sensitivity analysis conducted as part a global lake methanogenesis study, Ea' values were changed randomly in each grid cell within a qualified uncertainty range, and global patterns in J(T) did not change significantly (Jansen et al., 2022). This suggests that Ea' values are robust over space and time within the expected warming range, though more research on this topic is clearly warranted. "
